# Spectroscopic characterization and in vitro studies of biological activity of bradykinin derivatives

**DOI:** 10.1038/s41598-022-23448-7

**Published:** 2022-11-08

**Authors:** Edyta Proniewicz, Grzegorz Burnat, Helena Domin, Emilia Iłowska, Adam Roman, Adam Prahl

**Affiliations:** 1 Faculty of Foundry Engineering, AGH University of Science and Technology, 30-059 Krakow, Poland; 2 Maj Institute of Pharmacology, Polish Academy of Sciences, Department of Neurobiology, 12 Smętna Street, 31-343 Kraków, Poland; 3 Faculty of Chemistry, University of Gdansk, Wita Stwosza 63, 80-308 Gdansk, Poland; 4 Maj Institute of Pharmacology, Polish Academy of Sciences, Department of Brain Biochemistry, 12 Smętna Street, 31-343 Kraków, Poland

**Keywords:** Biochemistry, Cancer, Molecular medicine, Chemistry, Nanoscience and technology

## Abstract

Eleven multiple analogs of bradykinin—a peptide that is a natural ligand of B1 and B2 receptors but does not bind or activate the B1 receptor unless Arg^9^ is removed from the sequence by the action of carboxypeptidase N—were synthesized. Their biological activity was examined on T-REx cell lines expressing B1 or B2 receptors using the intracellular IP1 assay. The mRNA expression of B1R and B2R in the lysate of tumor cell lines, e.g., U87-MG (human astrocytoma), SHP-77 (human small cell lung cancer), and H4 (human brain glioma), was determined. For five B1R antagonists, adsorption at the liquid/solid interface (Au nanoparticles (AuNPs) served as the solid surface) was discussed in terms of the vibrations of molecular fragments (structural factors) responsible for the biological properties of these analogs.

## Introduction

Bradykinin (BK) (Table 1) belongs to the kinin protein group and influences a wide range of physiological responses via two metabotropic G protein-coupled receptors (GPCRs) known as B1 (B1R) and B2 (B2R). B2Rs are constitutively expressed in most cell types, whereas B1Rs are present only in tissue inflammation^1^. Activation of B2R causes slow, sustained smooth muscle contraction, alteration of epithelial cell ion secretion, increased vascular permeability, increased mucosal secretion, release of cytokines from leukocytes, stimulation of sensory neurons, and production of eicosanoids from a variety of cell types^2,3^. BK has been linked with a variety of diseases, including chronic pain, sepsis, asthma, rheumatoid arthritis, pancreatitis, and several other inflammatory diseases. BK is also considered a growth factor involved in the development and progression of cancer^4^. However, the mechanism of the tumor growth-promoting effect of this kinin is still unknown. GPCRs can be blocked by GPCR antagonists, which effectively compete with native peptides. Therefore, GPCR antagonists are an effective chemotherapeutic and diagnostic strategy in drug development^5,6^. In this context, several studies continue to focus on the development of mutant analogs that have both much higher affinity and antagonistic properties and weaker agonistic properties of these receptors. Because of these properties, specifically modified analogs can effectively compete with the native peptide; when they bind preferentially to GPCRs, they block these receptors from acting. Since clinical studies have shown that the main actions of BK are B2R functions, most attempts to synthesize effective BK antagonists have focused on analogs known as B2 bradykinin antagonists^7,8^. The search for B1R antagonists is also important because B1Rs are potential targets for drug development in pain processes^9^.

**Table 1:** Sequences of studied bradykinin analogs.

Abbrev.	Peptides’ sequence										
	0’	0	1	2	3	4	5	6	7	8	9
BK			Arg	Pro	Pro	Gly	Phe	Ser	Pro	Phe	Arg
BK1		**Aaa**	Arg	Pro	Pro	Gly	Phe	Ser	Pro	Phe	Arg
BK2		**Aca**	Arg	Pro	Pro	Gly	Phe	Ser	Pro	Phe	Arg
BK3		**D-Arg**	Arg	Pro	**Hyp**	Gly	**Thi**	Ser	**D-Phe**	**L-Pip**	Arg
BK4	**Aaa**	**D-Arg**	Arg	Pro	**Hyp**	Gly	**Thi**	Ser	**D-Phe**	**L-Pip**	Arg
BK5		**D-Arg**	Arg	Pro	**Hyp**	Gly	**Thi**	Ser	**D-Phe**	**D-Pip**	Arg
BK6	**Aaa**	**D-Arg**	Arg	Pro	**Hyp**	Gly	**Thi**	Ser	**D-Phe**	**D-Pip**	Arg
BK7	**Aaa**	**D-Arg**	Arg	Pro	**Hyp**	Gly	**Thi**	Ser	**D-Phe**	**Thi**	Arg
BK8		**D-Arg**	Arg	Pro	**Hyp**	Gly	**Thi**	Ser	**L-Pip**	**Thi**	Arg
BK9	**Aaa**	**D-Arg**	Arg	Pro	**Hyp**	Gly	**Thi**	Ser	**L-Pip**	**Thi**	Arg
BK10		**D-Arg**	Arg	Pro	**Hyp**	Gly	**Thi**	Ser	**D-Pip**	**Thi**	Arg
BK11	**Aaa**	**D-Arg**	Arg	Pro	**Hyp**	Gly	**Thi**	Ser	**D-Pip**	**Thi**	Arg

Aaa—[(3 s,5 s,7 s)-adamantan-1-yl]acetic acid, Aca -(3 s,5 s,7 s)-adamantane-1-carboxylic acid, Thi – thienylalanine, Pip—piperidine-2-carboxylic acid, and Hyp—hydroxyproline.

Understanding the effects of replacing naturally occurring amino acids with unnatural ones on peptide structure is critical to explaining and determining the role of individual amino acids in the physiological effects of BK. In this context, a number of analogs have been developed^8–18^. For example, it was discovered that the substitution of the L-proline residue at position 7 (Pro^7^) with D-phenylalanine ([D-Phe^7^]BK) is the key structural change in the sequence BK that alters the steric position of the *C*-terminal dipeptide Phe^8^–Arg^9^ and leads to antagonistic B2R activity^8,10^. The addition of a D-arginine at the *N*-terminus (D-Arg^0^) and the replacement of Pro^3^ with L-hydroxyproline (Hyp) increased the potency of the antagonist D-Arg^0^[Hyp^3^,D-Phe^7^]BK^10^. Replacement of two L-Phe residues at positions 5 and 8 with an unnatural amino acid that alters aromaticity in the *C*-termini has been shown to result in a partial agonist [Thi^5,8^]BK^11^. The addition of conformational constraints to the structure by replacing Phe^8^ with a piperidine-2-acetic acid (Pip) group results in a compound with increased potency in vitro and metabolic stability towards B1R^13^. Removal of the *C*-terminal Arg^9^ significantly decreases affinity for B2R. Acylation of the *N*-terminus with various bulky alkyl groups consistently improves antagonistic potency in rat blood pressure assays^12^. Peptides with a suitable achiral, nonaromatic, conformationally bound amino acid, such as 1-aminocyclohexane-1-carboxylic acid (Acc) at position 7, can also have an antagonistic effect on B2R^14,15^. Substitution of five non-proteinogenic amino acids yields the BK analog icatibant ([D-Arg^0^,Hyp^3^,Thi^5^,D-Tic^7^,Oic^8^,desArg^9^]BK), which is a potent, specific and selective peptidomimetic B2R antagonist^16^. Icatibant has also been used to show that B1Rs increase in neuronal cells of STZ-diabetic rats. In pioneering work, D. Regoli and colleagues and F. Marceau also identified and characterized novel multimutated derivatives of [desArg^9^]BK that act as kinin B1 receptor agonists^17,18^.

Despite the great interest in BK and its antagonists, which are selective for a single receptor class and represent an extremely important tool for elucidating the physiological and pathological roles played by naturally occurring peptides and their receptors, there is still a great need for research on BK analogs with antagonistic properties. The studies presented in this paper are an integral part of this scientific problem and are devoted to several analogs of BK (Table 1) and the determination of their biological activity using cell lines overexpressing B1R and B2R, as well as the intracellular inositol monophosphate (IP1) assay.

The studies presented here also describe peptide structures and adsorption changes resulting from modifying the peptide chain. These issues need to be addressed to better understand the interaction mechanism between BK and the receptors and are critical for the development of new BK antagonists with increased affinity for the receptors. Surface-enhanced Raman scattering (SERS) has shown promise in structural-activity studies to predict the biochemical activity of some neuropeptides^19–21^. For example, SERS has been used to determine the adsorbed molecular structures and the changes in these structures and adsorption resulting from substitution of natural amino acids by synthetic amino acids of bombesin (BN) and its analogs deposited on the Ag surface^19^. In the BN fragments, the relative efficacy of inhibiting the binding of ^125^I-[Tyr^4^]BN to rat pancreatic acini cells was correlated with the behavior of the amide binding on the Ag surface, whereas the contribution of structural components to the ability to interact with the GPCR was correlated with the SERS patterns. In other words, SERS enabled the identification of important amino acid residues involved in substrate-receptor interactions in systems where biological studies are difficult or do not clearly identify the molecular fragments responsible for the biological activity of the peptide.

SERS is a method for studying structure, molecular interactions, and conformational changes based on their vibrational fingerprint^22^. It uses highly concentrated fields in the vicinity of resonantly excited plasmonic structures to obtain a signal enhancement of 10^3^ to 10^10^ compared to the Raman signal^23^. In SERS, the energy of the incident radiation is very low and the risk of sample destruction or damage is low, so it can be successfully used to study biological material. SERS uses nanostructures of Ag^24^, Au^25^, and, less commonly, Cu^26^, or other metals^27,28^. Metal sols are the most biologically important nanostructures because they ensure low dispersion of nanoparticle diameter in the sol, do not require strict topological control, and provide strong enhancement that decreases dramatically with a continuous metal layer^29^. In humid air, Cu, Ag, and Au slowly corrode and darken to form a metal oxide layer. Corrosion of metals in the human body is always accompanied by a gradual and slow release of metal ions into the body^30^. Wataha et al. examined various Au alloys for the release of metal ions into the cell culture medium and found that Au ions generally do not dissolve in the medium, but Ag and Cu ions do^31^. For this reason, we chose colloidal Au for our study.

## Results and discussion

### Biological activity studies

**Figure 1:**
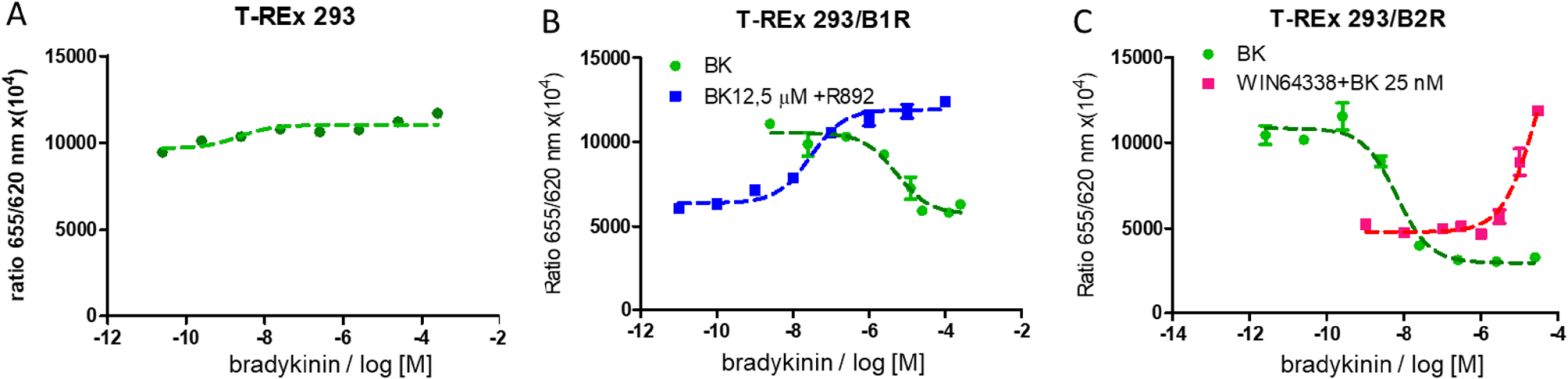
Influence of BK on IP1 levels in the mock T-REx 293 cell line. There is no functional activity of a BK receptor in this cell line because it is not expressed (**A**) and the effect of BK on T-REx 293 cells expressing B1R (**B**) or B2R (**C**). A dose-dependent increase in intracellular IP1 level was observed in presence of BK. In addition, specific antagonist activity for B1R and B2R was examined in the presence of BK, corresponding to EC_80_ for each receptor (12.5 µM for B1R and 25 nM for B2R).

Mock cells without transfection of bradykinin receptors were first used for the functional assay. As shown in Fig. 1A, the T-REx 293 cell lines did not respond to various doses of BK in the IP1 assay. Next, a cell line expressing one of the BK receptors was analyzed. Cells were exposed to different doses of BK and the influence of the selected reference antagonists R 892 (B1R antagonist) and WIN 64338 (B2R antagonist) was checked (Fig. 1B,C). The T-REx 293/B1R cell line increased intracellular IP1 levels in a dose-dependent manner. The half-maximal effective concentration (EC_50_) for B1R was calculated to be 1305 nM (SD ± 675 nM). The B1R antagonist R 892 decreased IP1 levels in a dose-dependent manner in the presence of concentration EC_80_ BK (1250 nM). The EC_50_ value for R 892 was calculated to be 69 nM (SD ± 66 nM) (Fig. 1B). The BK showed higher efficacy for B2R EC_50_ 8 nM (SD ± 5 nM) in the T-REx 293 cell lines. The B2R antagonist WIN 64338 showed a dose-dependent inhibitory effect in the presence of BK 25 nM, corresponding to EC_80_ (Fig. 1C).

**Figure 2:**
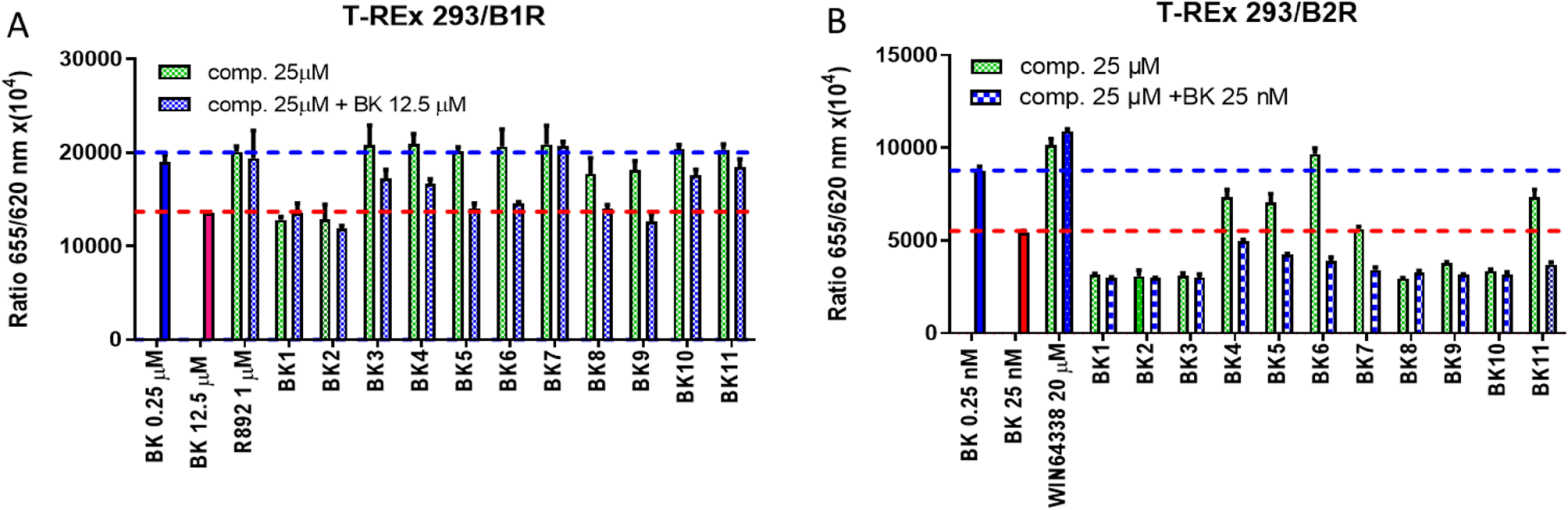
The activity of BK derivatives in the screening assay: (**A**) B1R and (**B**) B2R. The blue dashed line represents the lowest BK concentration that does not work, and the red line corresponds to the EC_80_ value for a given receptor. Agonistic effects were examined by treating cells with the compounds alone (green bars) or with BK in EC_80_ to examine their inhibitory effects (red bars).

Eleven derivatives of BK were analyzed in the screening procedure using both cell lines. The compounds were administered alone or in the presence of BK, corresponding to the EC_80_ of the antagonist (Fig. 2). For B1R, compounds BK1 and BK2 showed agonistic activity alone (Fig. 2A). Compounds BK5/6/8 and BK9 were neutral to receptor activity but did not abolish bradykinin action. An antagonistic effect in the presence of BK was observed for compounds BK3/4/7/10 and BK11.

In screening experiments with the B2R cell line, compounds BK4, BK5, BK6, and BK11 showed a mild agonistic effect (Fig. 2B). The derivative BK6 has no agonistic effect, but also does not reverse the effect of BK. The agonistic effect was significantly stronger for compounds BK1/2/3/7/8/9 and BK10.

**Figure 3:**
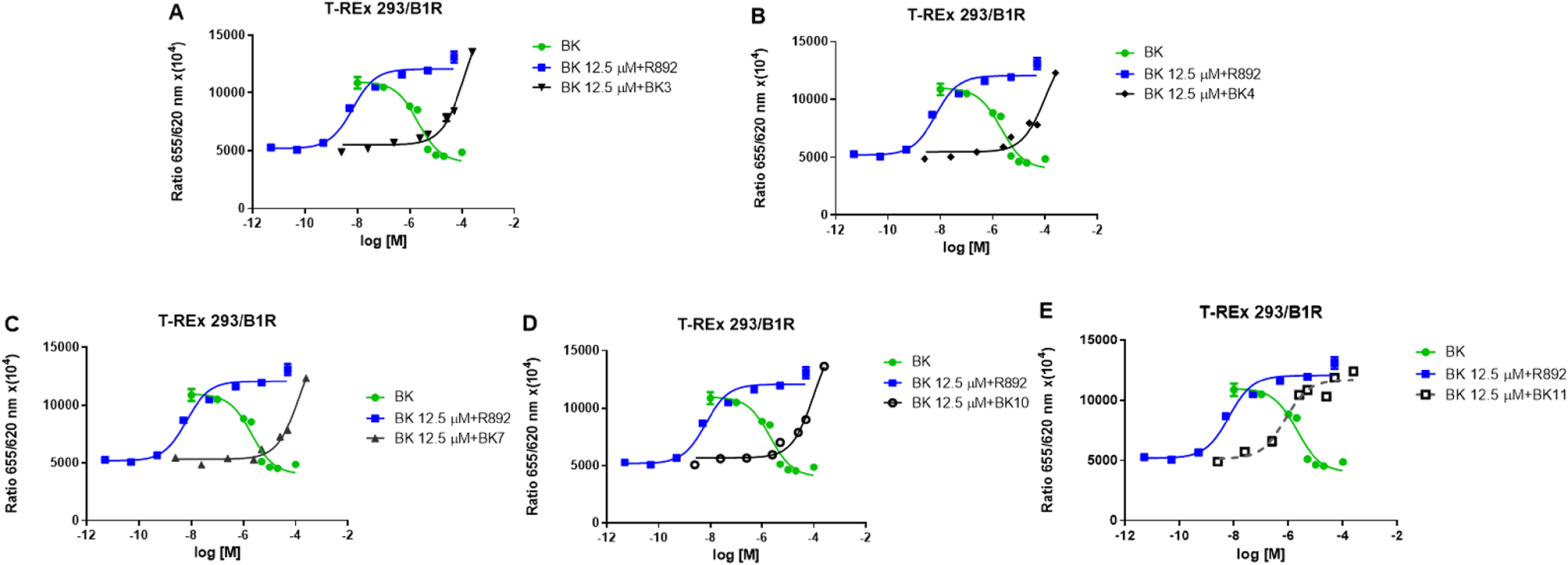
The pharmacodynamic effects of compounds showing inhibitory activity in the B1R screening assay. R892, the B1R antagonist, was used as the reference compound (blue line).

For all compounds that showed an antagonistic effect in the presence of bradykinin in the T-REx 293/B1R cell line, we attempted to determine the IC_50_ in the concentration range 2.5 × 10^–9^–2.5 × 10^–4^ M (Fig. 3). We succeeded in determining this parameter for compound BK11 (Fig. 3E), and it was 1186 nM (SD ± 817 nM). Other bradykinin derivatives were active only at the highest concentration between 25 µM and 250 µM, and the sigmoidal shape of the dose–response curve was not observed for these compounds because there was no saturation effect.

**Figure 4:**
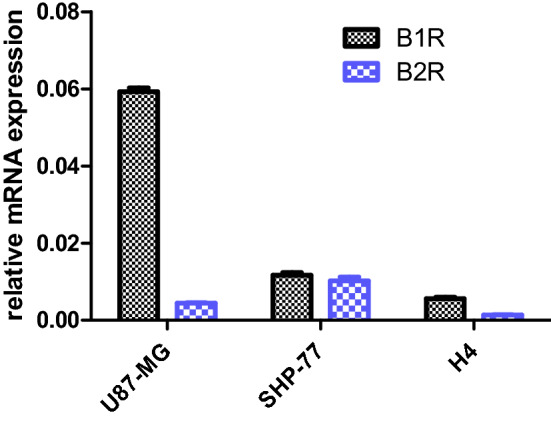
mRNA expression of B1R and B2R in the lysate of cancer cell lines: U87-MG, SHP-77, and H4.

The expression of B1R and B2R was examined in some tumor cell lines, such as U87-MG, SHP-77, and H4 using a qRT-PCR method. As shown in Fig. 4, mRNA expression for both receptors was detected in all cell lines. B1R mRNA was greatest in the U87-MG cell line and lowest in the H4 cells. In general, the B2 receptor mRNA was lower compared with that of B1R. B2R mRNA was most abundant in SHP-77 and least abundant in the H4 cell line.

In further experiments, the U87-MG, SHP-77, and H4 cell lines were used for the functional assay with BK and the reference B1R antagonist. As shown in Fig. 13S in Supplementary File, all three cell lines expressed the functional B1R protein and its activity according to the mRNA level shown in Fig. 4. The obtained results are consistent with previous findings showing the expression of functional B1R and B2R genes and proteins in human glioblastoma cells U87-MG^32,33^. In other studies, cellular expression of B1 and B2 receptors was detected in five different subtypes of human lung carcinomas (adenocarcinoma, squamous cell carcinoma, large cell carcinoma, small cell carcinoma, and carcinoid tumors)^34^. In addition, B2R has also been characterized in the cell line SHP-77^35^. However, to our knowledge, this is the first time that we have detected B1R and B2R gene expression in H4 cells of human brain neuroglioma and B1R in SHP-77 cells of human lung carcinoma. Interestingly, both U87-MG and H4 cell lines expressed higher levels of B1R mRNA compared with B2R, which could be explained by the inducible property of B1R under abnormal conditions, such as cancer^36^.

The cytotoxic potential of B1R antagonists such as BK3, BK4, BK7, BK10, and BK11 in H4, U87-MG, and SHP-77 cells was evaluated using the MTT assay, the most commonly used assay to evaluate the cytotoxic activity of the compounds. Cisplatin, a cytostatic drug commonly used for cancer chemotherapy, was used as a reference compound. Novel B1R antagonists exhibited varying levels of cytotoxic activity across cell lines and concentrations (Table 2). The obtained results indicate that the B1R derivatives studied may have anti-cancer properties in cell lines H4, U87-MG, and SHP-77.

**Table 2:** The effects of B1R antagonists (BK7, BK3, BK4, BK10, and BK11) and cisplatin on cancer cell lines (H4, U87-MG, and SHP-77) using MTT assay.

	H4	U87-MG	SHP-77
Control	99.999 ± 8.74	99.999 ± 5.246	100.000 ± 3.402
Cisplatin 25 µM	25.389 ± 3.71****	50.357 ± 4.97****	53.631 ± 12.052****
Cisplatin 100 µM	5.809 ± 1.488****	32.63 ± 6.934****	37.989 ± 11.626****
**BK7** 100 µM	97.73 ± 10.825	73.685 ± 12.815	86.685 ± 3.392
**BK3** 25 µM	83.049 ± 5.547	71.45 ± 7.763	87.804 ± 2.366
**BK3** 100 µM	70.937 ± 6.678	67.69 ± 6.873*	69.182 ± 5.170*
**BK4** 25 µM	74.075 ± 11.923	65.679 ± 5.21*	72.081 ± 5.974*
**BK4** 100 µM	50.773 ± 8.516***	59.113 ± 7.61***	52.663 ± 2.26****
**BK10** 25 µM	74.264 ± 11.923	79.495 ± 3.125	76.587 ± 5.974
**BK10** 100 µM	60.421 ± 3.769**	68.451 ± 4.128*	52.663 ± 2.26*
**BK11** 25 µM	94.235 ± 3.538	96.125 ± 1.241	96.421 ± 3.474
**BK11** 100 µM	65.668 ± 3.305*	81.07 ± 4.542	85.391 ± 5.646

Data were normalized to absorbance in the control group (100%) and are expressed as a percentage of control and presented as mean ± SEM obtained from *n* = 3 wells per experiment from 2–3 independent experiments. ****p < 0.0001; ***p < 0.001; **p < 0.01; *p < 0.05 (one-tailed ANOVA and Tukey post hoc test).

To verify whether the cytotoxic effects of the new B1R antagonists are mediated by apoptosis, the activation level of caspase-3, a key enzyme in the apoptosis process, and PARP protein, the caspase-3 substrate, were determined by Western blot analysis.

**Figure 5:**
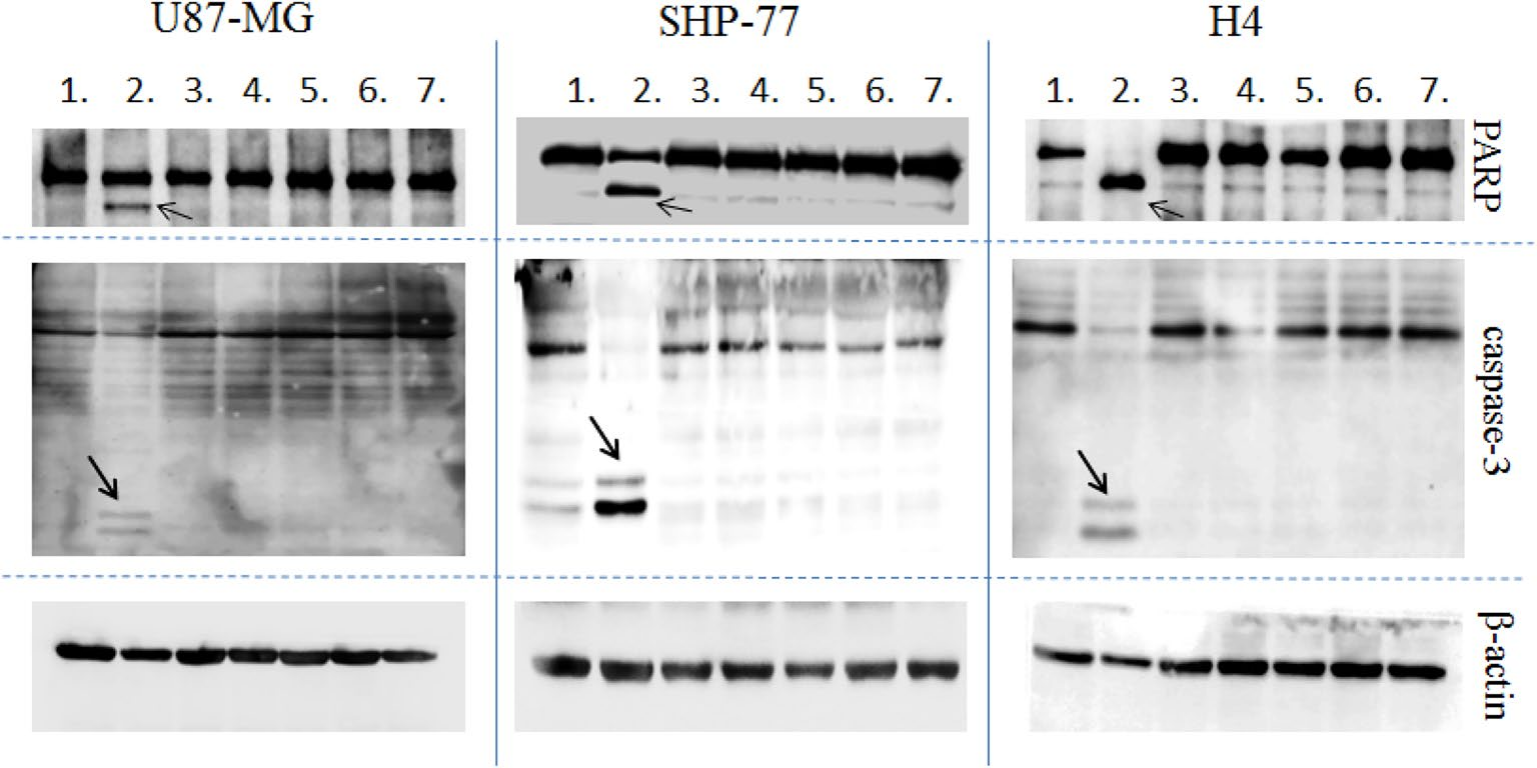
The effects of B1R antagonists and cisplatin on the expression levels of caspase-3 and PARP cleavage in cell lines U87-MG, SHP-77, and H4 were determined by Western blotting. **1.** control cells; **2.** cisplatin (25 μM); **3.** BK7 (50 μM); **4.** BK3 (50 μM); **5.** BK4 (50 μM); **6.** BK10 (50 µM); **7.** BK11 (50 μM). Arrows indicate the induction of caspase-3 and processed PARP fragments in cells treated with cisplatin as a positive control of apoptosis. After 24 h of cell treatment, no induction of caspase-3 and PARP is seen compared with untreated control cells. Representative Western blot images showing expression of β-actin were used as a reference protein. Each picture represents a vertically cut part of nitrocellulose membranes. The membranes were cut before antibody application, considering the size margin according to the size of the molecular weight marker.

Cisplatin at 25 µM was used to induce control apoptosis. No significant change in the amount of cleaved PARP or caspase-3 activation was observed after B1R antagonist treatment compared with untreated cells (Fig. 5). These results suggest that the apoptotic caspase-3/PARP pathway is not involved in the cytotoxic activity of the B1R antagonists studied, which may be due to the low activity of the antagonists.

Our results are consistent with previous data showing that BK inhibitors can modulate cancer growth^37,38^, but to our knowledge, we have presented for the first time new results describing the cytotoxic effects of B1R antagonists in human brain neuroglioma cells H4 and human lung carcinoma cells SHP-77. We also found that B1R antagonists reduce the viability of glioma cells in human U87-MG glioblastoma astrocytoma cells. These results are in addition to previous literature evidence that B1R antagonists have cytotoxic effects on other glioblastoma cell lines such as U-138MG and U-251MG^39^.

### Analysis of SERS spectra

**Figure 6:**
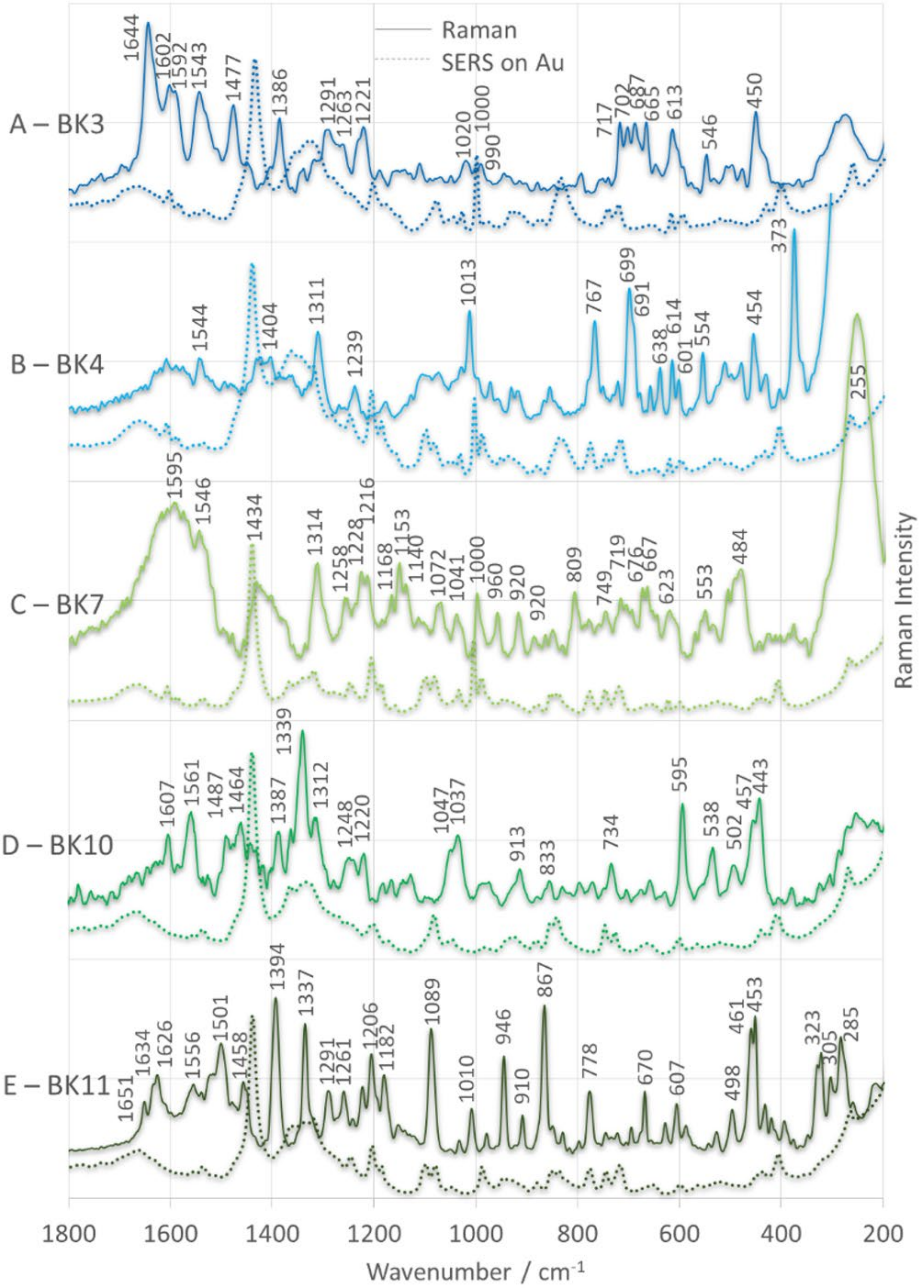
Raman (dashed line traces) and SERS (solid line traces) spectra of BK analogs adsorbed on AuNPs.

Five of the eleven analogs of BK, i.e., BK3, BK4, BK7, BK10, and BK11, have antagonistic properties for B1R in the presence of bradykinin in the T-REx 293/B1R cell line (R892 >  > BK11 >  > BK10 = BK7 = BK4 = BK3), but none for B2R in the presence of bradykinin in the T-REx 293/BK2R cell line. Figure 6 shows the SERS spectra (solid lines) for these analogs adsorbed on the surface of AuNPs. This figure also includes the corresponding Raman spectra (dashed lines) to highlight the differences (in terms of band enhancement, wavenumber, and half-width at band maximum (fwhm)) between the SERS and Raman spectra, which are necessary to properly describe adsorption.

The antagonistic property of BK11 towards B1R in the presence of native BK is much stronger than for other analogs. Taking into account the common mutations in the structure of all five peptides (D-Arg^0^, Hyp^3^, and Thi^5^), it appears that the combination of modifications in the D-Pip^7^ and Thi^8^ positions with Aaa^0'^ produces an antagonistic effect. For this reason, the discussion focuses on the analysis of the spectra in terms of the vibrations of the molecular fragments (structural factors) responsible for the biological properties of these analogs.

Following the work of Fang et. all, who showed the results for piperidine adsorbed on the surface of colloidal Ag and an Ag electrode, the most intense SERS band at 1394 cm^–1^ (fwhm = 17 cm^–1^) (Fig. 6E) is due to vibrations of D-Pip^7,40^. Based on density functional theory calculations (DFT), they proposed that this band corresponds to the ρ_ω_(CH_2_) mode of –CH_2_– groups adjacent to the nitrogen atom. Based on the work of Selvaraj et al., the other two intense bands at 1337 (fwhm = 12 cm^−1^) and 1089 cm^−1^ (fwhm = 15 cm^−1^) in the spectrum in Fig. 6E are assigned to the ν(C–C) + ρ_τ/ω_(CH_2_) + ρ_b_(CH) and ρ_τ_(CH_2_) + ρ_b_(CN–) + ρ_b_(CH) + ρ_b_(ring) vibrations, respectively^41^. Knowing that (i) piperidine is saturated, which means that the only possible interaction of piperidine with the substrate surface is via its lone pair of electrons on the nitrogen atom, (ii) the selection rules of SERS state that the vibrations whose intensity comes from a large value of α_zz_ (z is the local surface normal) are the most intense in the SERS spectrum, and (iii) the other intense vibrations observed in the SERS spectrum of BK11 are the in-plane skeletal stretching (at 867 (fwhm = 12 cm^−1^) and 1010 cm^–1^ (fwhm = 10 cm^−1^)) and the twisting vibrations (at 946 (fwhm = 10 cm^−1^), 1182, and 1261 cm^–1^)^42^, we can assume that piperidine is adsorbed in the end-on geometry with the N-containing molecular fragment interacting with the surface of the AuNPs.

We can expect similar bands to appear in the SERS spectrum of a BK10 analog (Fig. 6D) that contains D-Pip^7^ in its sequence. In this peptide SERS spectrum, the band at 1387 cm^–1^ is relatively weakly enhanced and broadened (fwhm = 21 cm^−1^), whereas the band at 1339 cm^−1^ (fwhm = 17 cm^−1^) is the strongest in the spectrum (I_1387_/I_1339_ = 0.6 compared with I_1394_/I_1337_ = 1.1 for BK11). The other piperidine bands are absent or only weakly enhanced for BK11. Therefore, it can be assumed that the piperidine ring lies flat on the surface of the AuNPs. This can be confirmed by the bands at 1037 [ρ_ω_(CH_2_) + ρ_ipb_(CH) + ρ_b_(ring)], 1047 [ρ_ω_(CH_2_) + ρ_b_(ring)], 1317 [ρ_ω_(CH_2_)], and 1464 cm^−1^ [ρ_s_(CH_2_)]^20,41^, which are more intense in the SERS spectrum of BK10 than in the SERS spectrum of BK11.

In the sequence of each peptide there is a Thi residue at position 5. The analogs BK7, BK10, and BK11 also contain Thi at position 8. The vibrations characteristic of the 2-substituted thiophene ring can be assigned to the bands at 1501 (fwhm = 23 cm^−1^), 1458 (fwhm = 18 cm^−1^), 1206 (fwhm = 10 cm^−1^), 670 [ρ_oopb_(CH), fwhm = 8 cm^−1^], 630 [ρ_ipb_(ring) + ν(C–S), fwhm = 9 cm^−1^], 530 [ring deformation + ρ_oopb_(CH), fwhm = 11 cm^−1^)], 498 [ρ_ipb_(C–CH_2_)_subst_, fwhm = 12 cm^−1^], 461 (fwhm = 9 cm^−1^), and 453 cm^−1^ (fwhm = 10 cm^−1^) in the BK11 SERS spectrum^20,41,42^. The SERS signals at 1501 and 1458 cm^−1^ are due to the antisymmetric and symmetric ν(C_α_ = C_β_) modes, respectively, conjugated to δ(CH)^43^. A band at 1206 cm^−1^ is assigned to the ν(C_α_–C_α2_) ring mode^44^^.^ The bands at 461 and 453 cm^−1^ are due to ring deformation (torsion) coupled to ρ_oopb_(CH)^45^. All of these bands are smaller in intensity and width than the piperidine bands. However, two of them, at 1501 and 1206 cm^−1^, are the most intense vibrations of Thi and the bands with the fraction of C–S vibrations (at 747, 850, and 630 cm^−1^) have the lowest intensity. It is also worth noting that ν(Ag–S), which is expected in the wavenumber range of 180–250 cm^−1^ when the adsorbate from the first monolayer interacts directly with the metal surface by charge transfer from the adsorbate to the metal or vice versa, is not present in the SERS spectrum of BK11^46^. According to the surface selection rule of Moskovits et al., the above results indicate a nearly horizontal orientation of the plane of the thiophene ring near the AuNP surface, which means that the molecule interacts only weakly via the sulfur atom.

In the SERS spectrum of BK10 analog (Fig. 6D), the Thi bands are generally less intense than the corresponding SERS signals for BK11, except for the bands at 502, 457, and 443 cm^−1^. In addition, the band at 538 cm^−1^ is more intense and a new band appears at 595 cm^−1^ [ρ_ipb_(ring) + ν(C–S)]. Also, in BK7 (Fig. 6C), most of the Thi bands decrease in intensity compared to BK11, while the SERS signals at 1501 and 1458 cm^−1^ practically disappear, and the 255 cm^−1^ spectral feature, which is due to ν(Au–N), is the strongest band in the spectrum^47^. Moreover, in the low-energy region of the spectra (below 400 cm^−1^), where the ν(Au–S) vibrations are expected, three bands of intermediate intensity appear only for BK11 at 323, 305, and 285 cm^−1^. Comparing the data from the literature, the above bands can be assigned to different types of Au–S bond vibrations, i.e., radial Au–S bond stretching at 285 cm^−1^, tangential Au–S bond stretching at 323 cm^−1^, and Au–S–C stretching at 305 cm^−1^^48^.

## Conclusions

Eleven bradykinin derivatives were synthesized and analyzed in a screening procedure using the T-REx 293/B1R and T-REx 293/B2R cell lines. Five of the eleven BK-analogs, including BK3, BK4, BK7, BK10, and BK11, have antagonistic properties for B1R in the presence of bradykinin in the T-REx 293/B1R cell line (R892 >  > BK11 >  > BK10 = BK7 = BK4 = BK3), but none show antagonistic properties for B2R in the presence of bradykinin in the T-REx 293/BK2R cell line. The cytotoxic/antiproliferative effects of these five analogs in cancer cell lines, such as H4, U87-MG, and SHP-77 were determined. The results showed that the B1R antagonists had varying degrees of cytotoxic effects depending on the cell lines and concentrations. This suggests that these analogs may have anticancer properties in cancer cell lines. Thus, our in vitro results suggest that B1R may be a promising target for cancer therapy. However, further data on the use of compounds with high B1R antagonist activity are needed to clarify the involvement of this receptor in anticancer strategies.

All five antagonists were immobilized at the AuNPs/liquid interface, and adsorption was monitored using SERS. The SERS spectra were analyzed in terms of the vibrations of these molecular fragments (amino acid side chains) responsible for the biological activity. Analysis of the SERS spectra shows that the combination of modifications at the D-Pip^7^ and Thi^8^ positions with Aaa^0'^ (the analog BK11) leads to a skeletal structure in which the piperidine ring adopts an end-on orientation with respect to the surface of the AuNPs. At this orientation, the nitrogen free electron pair is directed toward this surface, and the thiophene ring, whose free electron pair interacts only weakly with the AuNPs, assumes a nearly horizontal position relative to this surface. None of the other bradykinin analogs studied exhibits such an adsorbed structure, suggesting that the specific interaction between D-Pip^7^ and Thi^8^ and B1R, as well as the AuNP surface, may be responsible for the antagonistic properties of BK11.

## Methods

### Peptide synthesis

Peptides were synthesized via the solid-phase method using the Fmoc/*t*Bu strategy as previously described^49^ with minor modifications and purified (see Supporting Information for details).

### Biological studies

#### Cell culture and transfection

Cell lines overexpressing human B1R and B2R were prepared by transfection of the T-REx 293 cell lines (Thermo Fisher Scientific). This is a medicated HEK 293 (human embryonic kidney 293) cell line that allows the expression of the gene of interest in the pcDNA5/FTR/TO vector after the administration of tetracycline. The presence of tetracycline releases the Tet repressor protein (TetR) from the operon site and allows transcription of the mRNA (for details, see Supporting Information)^50^. The cancer cell lines, such as glioblastoma astrocytoma U87-MG and small cell lung carcinoma SHP-77 were purchased in ECACC. The brain neuroglioma cell line H4 was derived from ATCC.

#### qRT-PCR

The level of mRNA expression for B1R and B2R in the cancer cell lines was measured. Total RNA from the cells was extracted using TRIzol reagent (Thermo Fisher Scientific) according to the instructions (see Supporting Information for further details). The expression of each gene was quantified using the comparative threshold cycle method (ΔCT).

#### Intracellular inositol monophosphate (IP-one) assay

Receptor function was determined by measuring intracellular inositol monophosphate concentration using the IP-one kit from Cis-bio according to the procedure described previously (see Supporting Information for details)^21^. R 892 and WIN 64,338 hydrochloride (both from Tocris) were used as reference antagonists for B1R and B2R, respectively^51^.

#### Cytotoxicity analysis

The colorimetric tetrazolium salt assay with 3-(3,4-dimethylthiazol-2-yl)-2,5-diphenyltetrazolium bromide (MTT) was used to evaluate the cytotoxicity potential of the antagonists BK in the cancer cell lines H4, U87-MG, and SHP-77 (see Supporting Information for details)^52^.

#### Western blot

Western blot analysis was performed to evaluate the potential mechanism of cytotoxic activity of the antagonists of BK in cancer cell lines H4, U87-MG, and SHP-77 (see Supporting Information for details). Detection was performed using Pierce ECL Western blotting substrate (Thermo Fisher Scientific) and a Syngene GeneGnomeXRQ chemiluminescence analysis system. Primary antibodies: β-actin (1:10,000, Sigma, A5441), caspase-3 (1:1000, Cell Signaling, #9662S), poly(ADP-ribose) polymerase (PARP, 1:1000, Cell Signaling, #9532). Secondary antibodies: anti-rabbit (1:2500, Promega W401B) and anti-mouse (1:2500, Promega W402B).

### Colloids

Three batches of colloidal Au solutions (20 nm, ~ 7.2 × 10^10^ particles/mL, reactant-free, polydispersity index (PDI) < 0.2, λ_max_ = 529–533 nm) were purchased from Merck (Poland).

### Raman and SERS measurements

Spectra were acquired using an InVia Raman spectrometer (Renishaw) with a CCD detector and a Leica microscope (50 × objective). The spectral resolution was set to 4 cm^−1^. A diode laser with a wavelength of 785 nm (20 mW) served as the excitation source. The typical exposure time was 40 s with five accumulations. The SERS spectra of a given peptide were almost identical. No spectral changes were observed that could be associated with decomposition of the sample.

## Supplementary Information

Supplementary Information.

## Data Availability

Data are available upon request (please contact the corresponding author: proniewi@agh.edu.pl).
