# Supplementary material for: Spectroscopic characterization and in vitro studies of biological activity of bradykinin derivatives
Source: Sci Rep. 2022 Nov 8;12:19015. doi: 10.1038/s41598-022-23448-7 (PMC9643506; doi:10.1038/s41598-022-23448-7)
Supplement: Supplementary file 1 — Supplementary Information. [file 41598_2022_23448_MOESM1_ESM.doc]

Spectroscopic characterization and *in vitro* studies of biological activity of bradykinin derivatives

Edyta Proniewicz^1,*^, Grzegorz Burnat^2^, Helena Domin^2^, Emilia Iłowska^3^, Adam Roman^4^, and Adam Prahl^3^

^1^Faculty of Foundry Engineering, AGH University of Science and Technology, 30-059 Krakow, Poland

^2^Maj Institute of Pharmacology, Polish Academy of Sciences, Department of Neurobiology, 31-343 Kraków, 12 Smętna Street, Poland

^3^Faculty of Chemistry, University of Gdansk, 80-308, Gdansk, Wita Stwosza 63, Poland

^4^Maj Institute of Pharmacology, Polish Academy of Sciences, Department of Brain Biochemistry, 31-343 Kraków, 12 Smętna Street, Poland

^*^corresponding author [proniewi@agh.edu.pl](mailto:proniewi@agh.edu.pl)

METHODS

**Peptide synthesis.** Peptides were synthesized via the solid-phase method using the Fmoc/*t*Bu strategy as previously described^49^ with minor modifications. Briefly, Fmoc-Arg-Wang Resin (GL Biochem, China) was used. Peptide chain extension was performed using an automated peptide synthesizer (Symphony, Gyros Protein Technologies, USA) in the reaction with a threefold excess of the respective Fmoc-amino acid in an equimolar mixture with *O*-(7-azabenzotriazol-1-yl)-*N,N,N’,N’*-tetramethyluronium hexafluorophosphate (HATU) and 1-hydroxy-7-azabenzotriazole (HOAt) and two equivalents of *N*-methylmorpholine (NMM). Fmoc-protected amino acids were purchased from commercial suppliers (Merck KGaA, Germany; GL Biochem, China). Peptide cleavage was performed using a standard mixture for 4 h. Crude peptides were precipitated with cold diethyl ether, centrifuged, dried, dissolved in water, and lyophilized overnight.

Peptides were purified using a preparative reversed-phase high-performance liquid chromatography system (RP-HPLC) (Shimadzu, Japan, and Waters, USA) with a Jupiter Proteo column (4 μM, 90 Å, 250 × 10 mm). The purity (> 95%) of the peptides was determined using an analytical RP-HPLC system (Shimadzu, Japan) with Jupiter Proteo column (4 μM, 90 Å; 250 × 4.6 mm), and the linear gradient of solution B in A from 1% to 80% in 30 minutes with a flow rate of 1 mL/min. The eluents used were: A – 0.1% aqueous solution of TFA and B – 80% solution of acetonitrile in aqueous 0.1% TFA (v/v). The mass spectra of the peptides were recorded using Bruker BIFLEX III and autoflexmaX MALDI TOF mass spectrometers (see Supporting Information – Analytical Data of the Synthesized Peptides).

**Cell culture and transfection.** Cell lines overexpressing human bradykinin receptors 1 and 2 were prepared by transfection of the T-REx 293 cell lines (Thermo Fisher Scientific). This is a medicated HEK 293 (human embryonic kidney 293) cell line that allows expression of the gene of interest in the pcDNA5/FTR/TO vector after administration of tetracycline. The presence of tetracycline releases the Tet repressor protein (TetR) from the operon site and allows transcription of the mRNA.^50^ The sequence of human B1 receptor GenBank ACC# AY275464 in pcDNA 3.1+ plasmids (UMR cDNA Resource Centre) was introduced into the T-REx 293 cell line using Lipofectamine Reagent 3000 (ThermoFisher Scientific). Antibiotic G-418 at a dose of 500 µg/mL was used to select the transfected cells. The B2 receptor sequence (GenBank ACC# AY275465; UMR cDNA Resource Centre) was originally located in pcDNA3.1+ and was subcloned into the vector pcDNA5/FTR/TO (ThermoFisher Scientific) using the restriction enzymes BamHI and XhoI. Cells were cotransfected with pcDNA5/FRT/TO-B2R and the pOG44 plasmid encoding Flp recombinase. Stably transfected cells were then prepared by antibiotic selection with hygromycin B 100 μg/mL. The expression of B2R was induced by the addition of tetracycline at a concentration of 0.75 μg/mL 24 hours before experiments in an FBS-free culture medium. Cells were grown under standard cell culture conditions (37°C, 5% CO_2_) in DMEM (Thermo Fisher Scientific) supplemented with 10% FBS (Thermo Fisher Scientific) and Glutamax (Thermo Fisher Scientific).

The cancer cell lines such as glioblastoma astrocytoma U87-MG and small cell lung carcinoma SHP-77 were purchased in ECACC. The brain neuroglioma cell line H4 originated from ATCC. U87-MG and H4 were cultured in DMEM supplemented with 10% FBS. The SHP-77 cell line was maintained in RPMI-1640 (ThermoFisher Scientific) and FBS 10%.

**qRT-PCR.** The level of mRNA expression for B1R and B2R in the cancer cell lines was measured. Total RNA from the cells was extracted using TRIzol reagent (Thermo Fisher Scientific) according to the instructions. The amount of RNA was determined using a DeNovix DS-11 spectrophotometer (DeNovix). For cDNA synthesis, 1 μg of total RNA was used. Reverse transcription was performed using a QuantiTect Reverse Transcription Kit (Qiagen) according to the instructions (the synthesis run was performed at 42°C for 20 min after an inactivation step at 95°C for 3 min). TaqMan real-time quantitative PCR amplification reactions were performed in a CFX96 Touch real-time PCR Detection System (Bio-Rad, USA). Real-time PCR was performed using the TaqMan Gene Expression Master Mix (Thermo Fisher Scientific). Briefly, a 15-μl reaction mixture consisted of 7.5 μl PCR Master Mix (Thermo Fisher Scientific), 0.75 μl TaqMan probe (Thermo Scientific, USA), 1 μl cDNA (diluted 1:10), and 5.75 μl RTPCR grade water (Thermo Fisher Scientific). All reactions were performed in triplicate. Thermal cycling conditions were as follows: 95°C for 10 minutes (Taq activation), 95°C for 15 seconds for denaturing, and 60°C for 1 minute for annealing and extension. The human TaqMan probes used in the study are B1R Hs00176121, B2R Hs00664201, and β-actin Hs99999903.

The expression of each gene was quantified using the comparative threshold cycle method (ΔCT).

**Intracellular inositol monophosphate (IP-one) assay.** Receptor function was determined by measuring the intracellular concentration of inositol monophosphate using the Cis-bio IP-one kit according to the procedure described previously^18^. Briefly, the cells were grown in an DMEN medium without FBS 24 hours prior to the trial. The cell pellet was suspended in Hanks-HEPES (130 mM NaCl, 5.4 mM KCl, 1.8 mM CaCl_2_, 0.8 mM MgSO_4_, 0.9 mM NaH_2_PO_4_, 20 mM HEPES, LiCl 50 mM, and 3.25 mM glucose; pH 7.4). Then, the cell suspension was incubated for 1h (37°C) on a 384 white low-volume plate (Grainer Bio-One) in the presence of an increased concentration of bradykinin or a 25 μM concentration of bradykinin analogs alone or in the presence of a bradykinin concentration equivalent to EC80 (total volume 10 μL; 1 x 10^4^ cells). The following 5 µL of the IP1-acceptor conjugate and 5 µL of the anti-IP1-donor conjugate have been added to each well. After one hour of incubation at RT, fluorescence was measured at 620 nm and 665 nm (Tecan; Infinite M1000). The results were calculated by multiplying the ratio (665 nm/620 nm) by 10^4^. The signal was inversely proportional to the IP1 concentration in the speciments. R 892 and WIN 64338 hydrochloride (both from Tocris) were used as reference antagonists for B1R and B2R, respectively^51^.

Each sample was prepared three times. Data were analyzed using GraphPad Prism version 5.04 for Windows (GraphPad software).

**Cytotoxicity analysis.** The colorimetric tetrazolium salt assay with 3-(3,4-dimethylthiazol-2-yl)-2,5-diphenyltetrazolium bromide (MTT) was used to evaluate the cytotoxicity/antiproliferative potential of the bradykinin analogs with antagonistic properties in cancer cell lines (H4, U87-MG, and SHP-77).

Cell lines were seeded at a concentration of 5000 cells/well (H4) and 10000 cells/well (U87-MG, SHP-77) in 96-well plates and incubated for 24 hours. These cell lines were treated with B1R antagonists (BK3/4/7/10/11) and cisplatin at concentrations of 25 and 100 µM. These compounds were dissolved in redistilled water and added to the cultures at a final concentration of 0.1%. Twenty-four hours after treatment with the drugs, the MTT assay was performed according to the procedure described previously.^52^ MTT solution was added to each well (at a final concentration of 0.15 mg/mL) and then incubated at 37°C for 30 minutes. Formosan crystals were resolved and measured at 570 nm using a 96-well microplate reader (Thermo Lab system Multiscan Spectrum). Data were normalized to absorbance in vehicle-treated cells (100%) and expressed as a percentage of control ± SEM determined from n = 5 wells per experiment from 2 to 3 separate experiments. Data were analyzed using GraphPad Prism 7.04 software. Statistical differences were calculated using a one-way analysis of variance (ANOVA) followed by Tukey’s posthoc test. Values of p < 0.05 were considered significant.

**Western blot.** Western blot analysis was performed to evaluate the potential mechanism of cytotoxic/antiproliferative activity of bradykinin analogs with antagonistic properties in cancer cell lines (H4, U87-MG, and SHP-77). Cell lines were seeded at a concentration of 2.50 x 10^5^ cells/well (H4) and 5 x 10^5^ cells/well (U87-MG and SHP-77) in 6-well plates and incubated for 24 hours. These cell lines were treated with B1R antagonists (BK3/4/7/10/11) at a concentration of 50 µM and with cisplatin at concentrations of 25 µM.

After growth in a medium containing tetracycline, stably transfected cells were washed twice with PBS and dissolved in a lysis buffer containing 20 mM Tris-HCl, 100 mM NaCl, 1 mM EDTA, 0.5% Triton X-100, and a protease inhibitor cocktail (ThermoFisher Scientific) for 20 min on ice. After centrifugation at 13,000 × g for 15 min at 4 °C, the supernatant was mixed with 2× Laemmli buffer and heated at 95 °C for 5 min. Subsequently, 15 µg of the proteins recovered from the cell lysates were separated on 8% sodium dodecyl sulfate-polyacrylamide electrophoresis gels (SDS-PAGE) and transferred to nitrocellulose membranes. After transfer, the membranes were incubated in TBST (Invitrogen) containing 5% nonfat milk (Bio-Rad) overnight at 4°C. Incubation was than performed with primary antibodies for caspase-3 (1:1000, CellSignaling #9662S), PARP (poly(ADP-ribose)polymerase-1) (1:1000, CellSignaling #9532), and β-actin (1:10000, A5441 Sigma) at RT for 1 hour. Membranes were then incubated with the secondary antibodies conjugated to horseradish peroxidase at room temperature for 1 h (W402B and W401B; Promega). Detection was performed using Pierce ECL Western blotting substrate (ThermoFisher Scientific) and a Syngene GeneGnomeXRQ chemiluminescence analysis system**.**

**Raman and SERS measurements.** Aqueous solutions of each peptide were prepared at a concentration of 10^-4^ mol/L and a pH of 7. 10 μL of the peptide solution was deposited onto 3 Au or Ag surfaces. The SERS spectra were collected 3 times at 3 different locations on each surface.

Nine samples of 40 μL nanoparticle solutions (three samples from three different batches of Au colloid) were mixed with 20 μL of each peptide solution. The final sample concentration was 3∙10^-5^ mol/L (no conventional Raman signal was observed at this sample concentration). The 20 μL peptide/sol mixture was applied to the glass plate, and the SERS were recorded (no measurements were made for the dried droplet).

Raman and SERS spectra were recorded using an InVia Raman Spectrometer (Renishaw) containing an air-cooled charge-coupled device (CCD) detector and a Leica microscope (50x objective). The spectral resolution was set to 4 cm^–1^. The 785 nm line of a diode laser was used as the excitation source. The laser power at the output was set at 20 mW. The typical exposure time for each Raman and SERS measurement was 40 s with five accumulations (series of 5 spectra, each accumulated 40 s = 200 s). The SERS spectra of a given peptide adsorbed on AgNPs or AuNPs from three different batches (bottles) were almost identical, except for small differences (up to 5%) in some band intensities. During the measurements, no spectral changes were observed that could be related to the sample decomposition.

Simplified Molecular Input Line Entry Specification

| Compound | SMILES |
| --- | --- |
| BK | O=C(N[C@H](Cc1ccccc1)C(=O)N[C@H](CCCNC(=N)N)C(=O)O)[C@H]1CCCN1C(=O)[C@@H](CO)NC(=O)[C@@H](Cc1ccccc1)NC(=O)CNC(=O)[C@H]1CCCN1C(=O)[C@H]1CCCN1C(=O)[C@@H](N)CCCNC(=N)N |
| BK1 | O=C(N[C@H](Cc1ccccc1)C(=O)N[C@H](CCCNC(=N)N)C(=O)O)[C@H]1CCCN1C(=O)[C@@H](CO)NC(=O)[C@@H](Cc1ccccc1)NC(=O)CNC(=O)[C@H]1CCCN1C(=O)[C@H]1CCCN1C(=O)[C@H](CCCNC(=N)N)NC(=O)C[C@]12C[C@H]3C[C@H](C[C@@H](C3)C1)C2 |
| BK2 | O=C(N[C@H](Cc1ccccc1)C(=O)N[C@H](CCCNC(=N)N)C(=O)O)[C@H]1CCCN1C(=O)[C@@H](CO)NC(=O)[C@@H](Cc1ccccc1)NC(=O)CNC(=O)[C@H]1CCCN1C(=O)[C@H]1CCCN1C(=O)[C@H](CCCNC(=N)N)NC(=O)C12C[C@H]3C[C@@H](C[C@@H](C3)C1)C2 |
| BK3 | O=C(O)[C@@H](CCCNC(=N)N)NC(=O)[C@H]1CCCCN1C(=O)[C@@H](Cc1ccccc1)NC(=O)[C@H](CO)NC(=O)[C@@H](NC(=O)CNC(=O)[C@H]1CC(O)CN1C(=O)[C@@H]1CCCN1C(=O)[C@@H](CCCNC(=N)N)NC(=O)[C@@H](N)CCCNC(=N)N)Cc1cccs1 |
| BK4 | O=C(O)[C@@H](CCCNC(=N)N)NC(=O)[C@H]1CCCCN1C(=O)[C@@H](Cc1ccccc1)NC(=O)[C@H](CO)NC(=O)[C@@H](NC(=O)CNC(=O)[C@H]1CC(O)CN1C(=O)[C@@H]1CCCN1C(=O)[C@@H](CCCNC(=N)N)NC(=O)[C@H](CCCNC(=N)N)NC(=O)C[C@]12CCC(CC1)CC2)Cc1cccs1 |
| BK5 | O=C(O)[C@@H](CCCNC(=N)N)NC(=O)[C@@H]1CCCCN1C(=O)[C@@H](Cc1ccccc1)NC(=O)[C@H](CO)NC(=O)[C@@H](NC(=O)CNC(=O)[C@H]1CC(O)CN1C(=O)[C@@H]1CCCN1C(=O)[C@@H](CCCNC(=N)N)NC(=O)[C@@H](N)CCCNC(=N)N)Cc1cccs1 |
| BK6 | O=C(O)[C@@H](CCCNC(=N)N)NC(=O)[C@@H]1CCCCN1C(=O)[C@@H](Cc1ccccc1)NC(=O)[C@H](CO)NC(=O)[C@@H](NC(=O)CNC(=O)[C@H]1CC(O)CN1C(=O)[C@@H]1CCCN1C(=O)[C@@H](CCCNC(=N)N)NC(=O)[C@H](CCCNC(=N)N)NC(=O)CC12CCC(CC1)CC2)Cc1cccs1 |
| BK7 | O=C(O)[C@H](CCCNC(=N)N)NC(=O)[C@H](Cc1cccs1)n12ccccc2C[C@H](NC(=O)[C@@H](CO)NC(=O)[C@@H](NC(=O)CNC(=O)[C@H]2CC(O)CN2C(=O)[C@@H]2CCCN2C(=O)[C@@H](CCCNC(=N)N)NC(=O)[C@H](CCCNC(N)=N)NC(=O)CC23CCC(CC2)CC3)Cc2cccs2)C1=O |
| BK8 | O=C(O)[C@@H](CCCNC(=N)N)NC(=O)[C@H](Cc1cccs1)NC(=O)C1CCCC[N@]1C(=O)[C@H](CO)NC(=O)[C@@H](NC(=O)CNC(=O)[C@@H]1CC(O)CN1C(=O)[C@H]1CCCN1C(=O)[C@H](CCCNC(=N)N)NC(=O)[C@H](N)CCCNC(=N)N)Cc1cccs1 |
| BK9 | O=C(O)[C@@H](CCCNC(=N)N)NC(=O)[C@H](Cc1cccs1)NC(=O)C1CCCC[N@@]1C(=O)[C@H](CO)NC(=O)[C@@H](NC(=O)CNC(=O)[C@@H]1CC(O)CN1C(=O)[C@H]1CCCN1C(=O)[C@H](CCCNC(=N)N)NC(=O)[C@@H](CCCNC(=N)N)NC(=O)CC12CCC(CC1)CC2)Cc1cccs1 |
| BK10 | O=C(O)[C@@H](CCCNC(=N)N)NC(=O)[C@H](Cc1cccs1)NC(=O)C1CCCC[N@@]1C(=O)[C@H](CO)NC(=O)[C@@H](NC(=O)CNC(=O)[C@@H]1CC(O)CN1C(=O)[C@H]1CCCN1C(=O)[C@H](CCCNC(=N)N)NC(=O)[C@H](N)CCCNC(=N)N)Cc1cccs1 |
| BK11 | O=C(O)[C@@H](CCCNC(=N)N)NC(=O)[C@H](Cc1cccs1)NC(=O)C1CCCC[N@]1C(=O)[C@H](CO)NC(=O)[C@@H](NC(=O)CNC(=O)[C@@H]1CC(O)CN1C(=O)[C@H]1CCCN1C(=O)[C@H](CCCNC(=N)N)NC(=O)[C@@H](CCCNC(=N)N)NC(=O)CC12CCC(CC1)CC2)Cc1cccs1 |

Analytical data of synthesized peptides


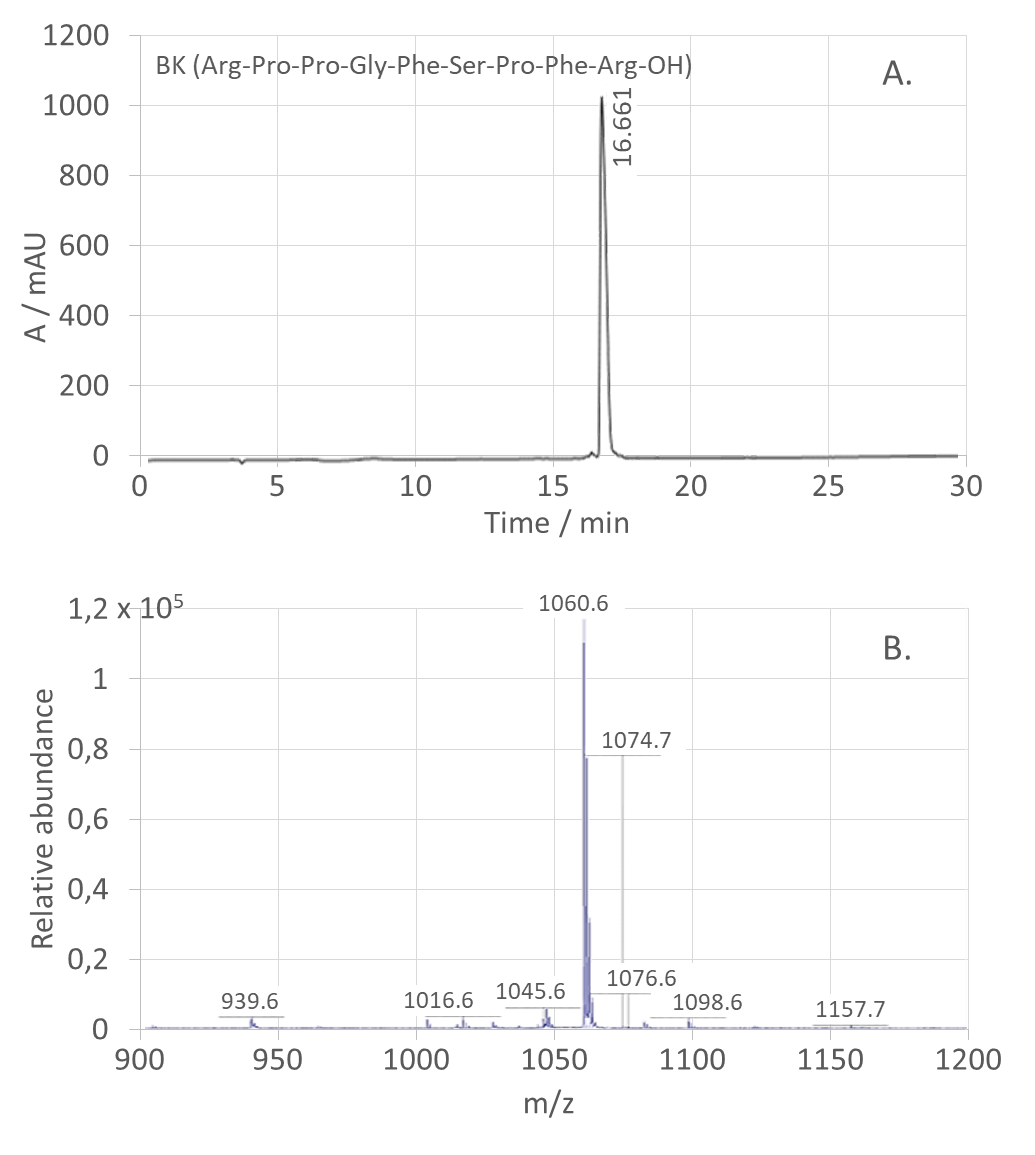


**Figure 1S.** The RP-HPLC analysis (A.) and mass spectrum ([M+H]^+^ calculated 1060.2, found 1060.6) (B.) of BK peptide.


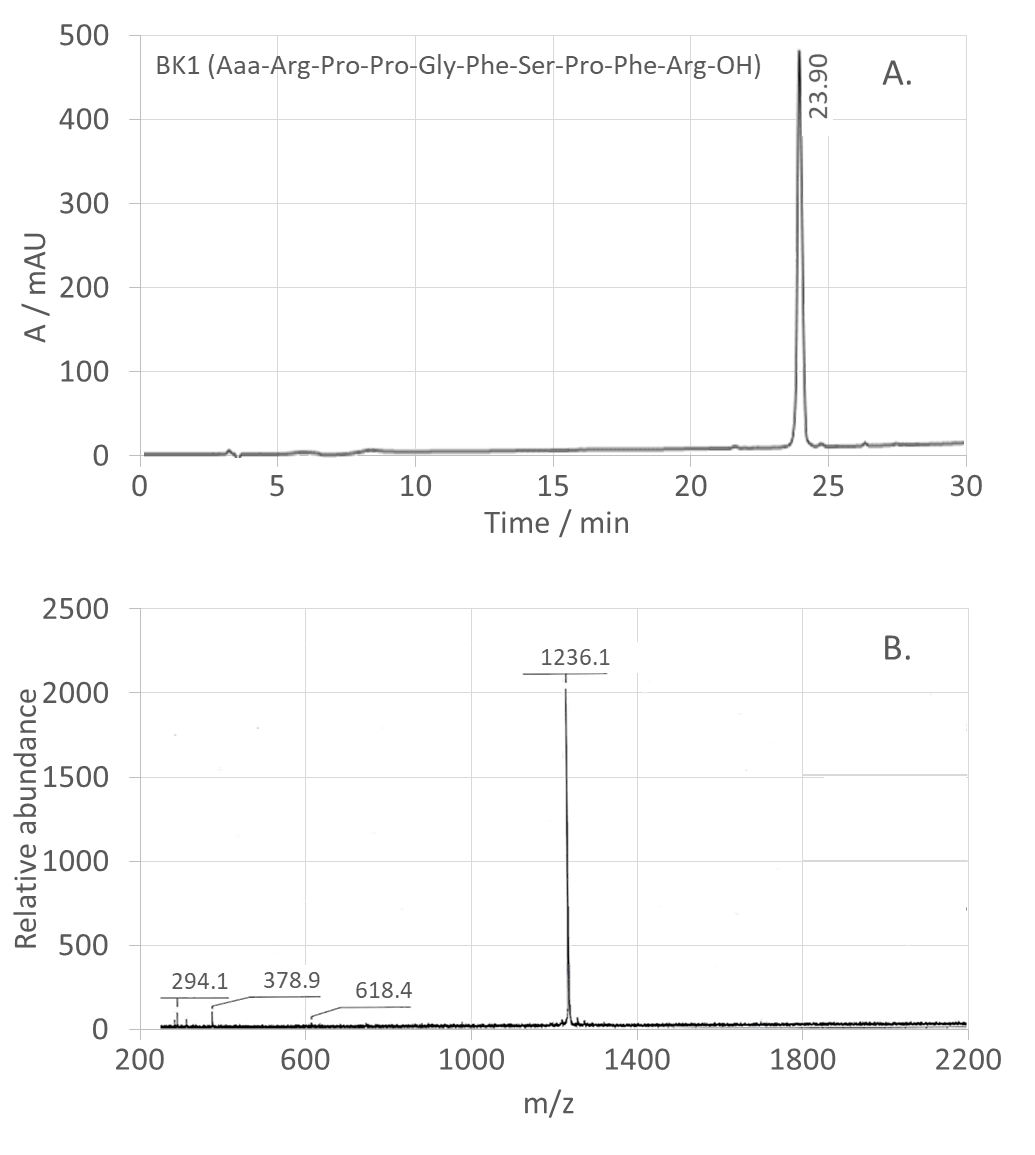


**Figure 2S.** The RP-HPLC analysis (A.) and mass spectrum ([M+H]^+^ calculated 1235.2, found 1236.1) (B.) of BK1 peptide.

**
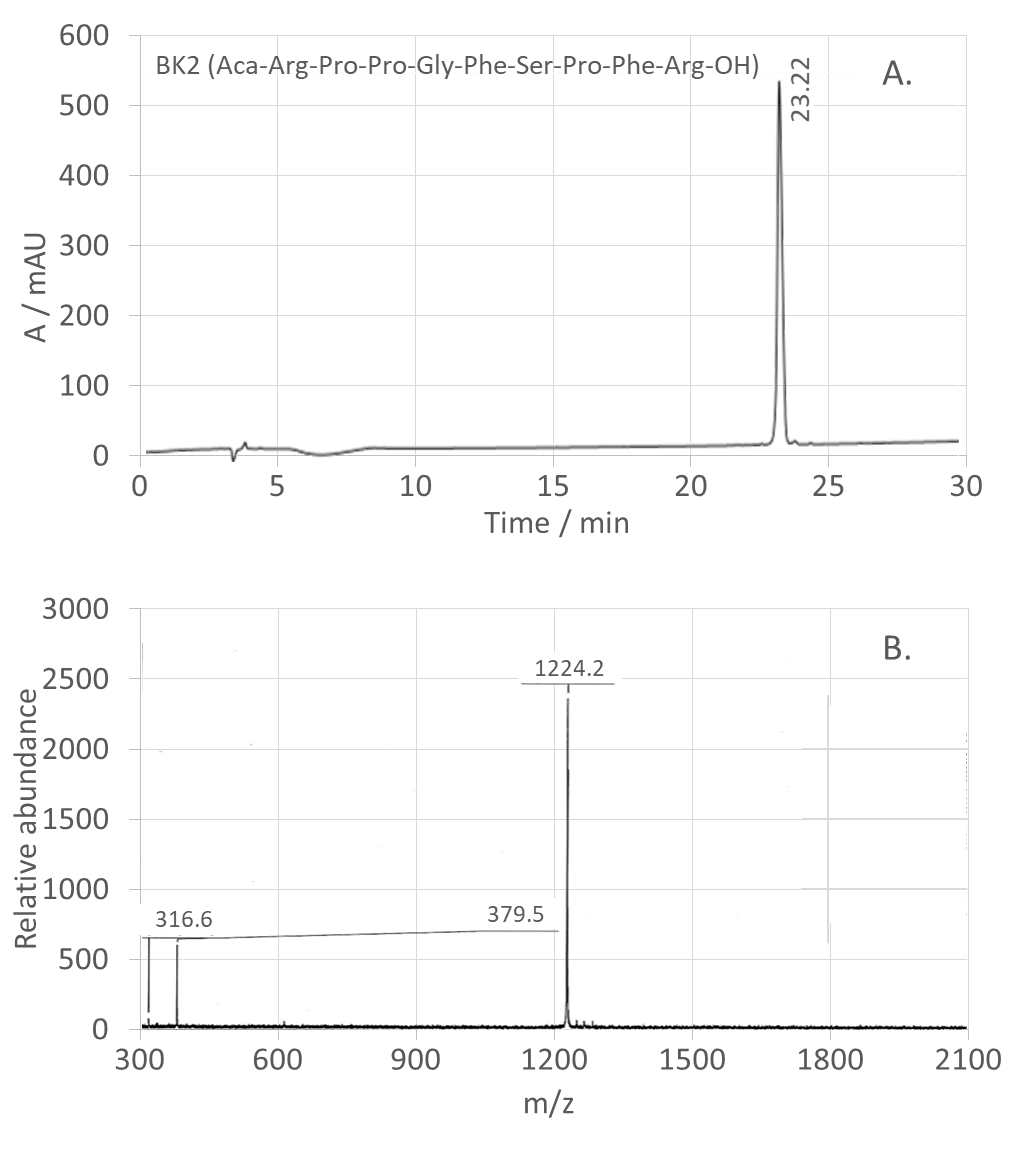
**

**Figure 3S.** The RP-HPLC analysis (A.) and mass spectrum ([M+H]^+^ calculated 1223.2, found 1224.2) (B.) of BK2 peptide.

**
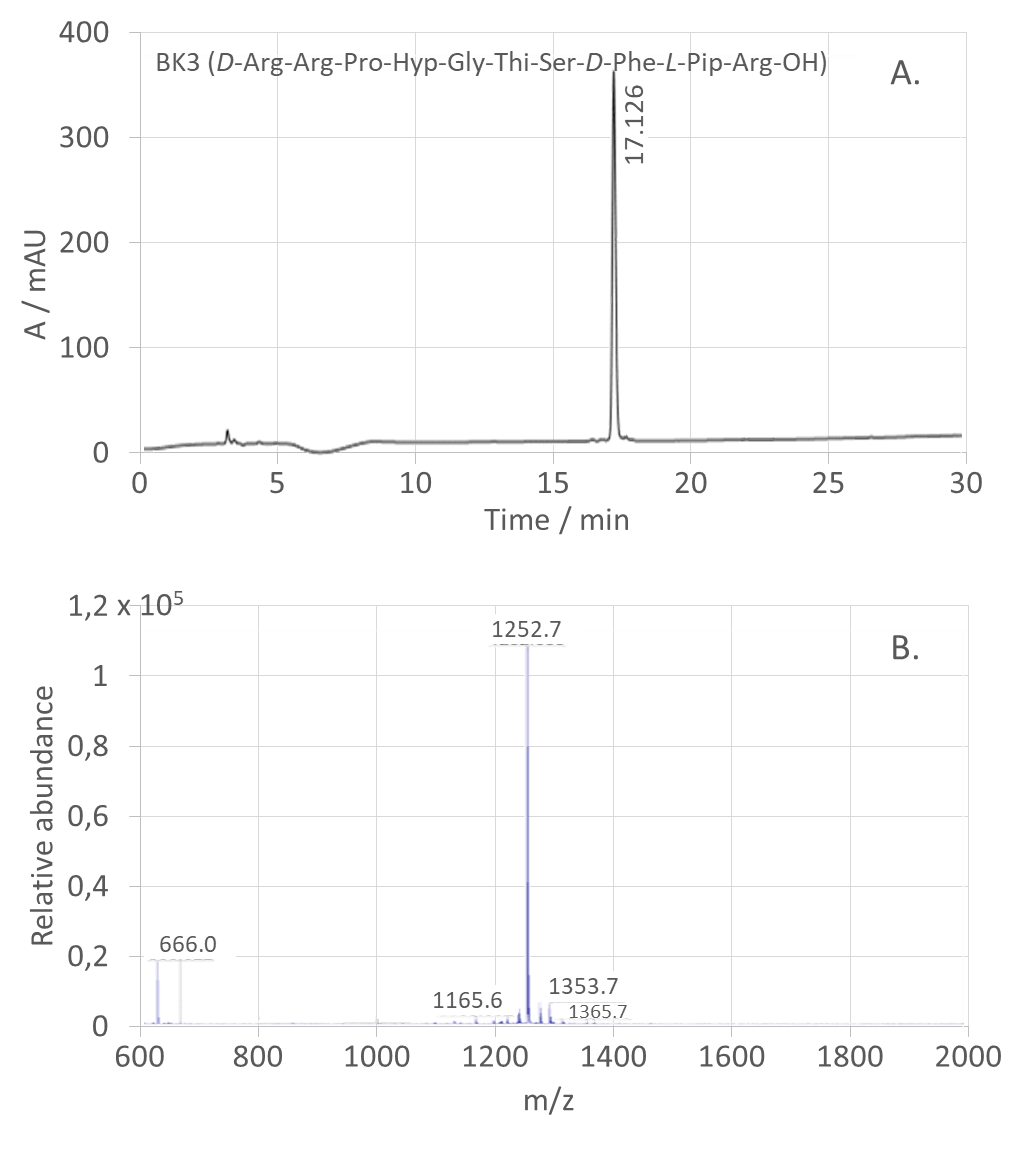
**

**Figure 4S.** The RP-HPLC analysis (A.) and mass spectrum ([M+H]^+^ calculated 1253.1, found 1252.6) (B.) of BK3 peptide.

***
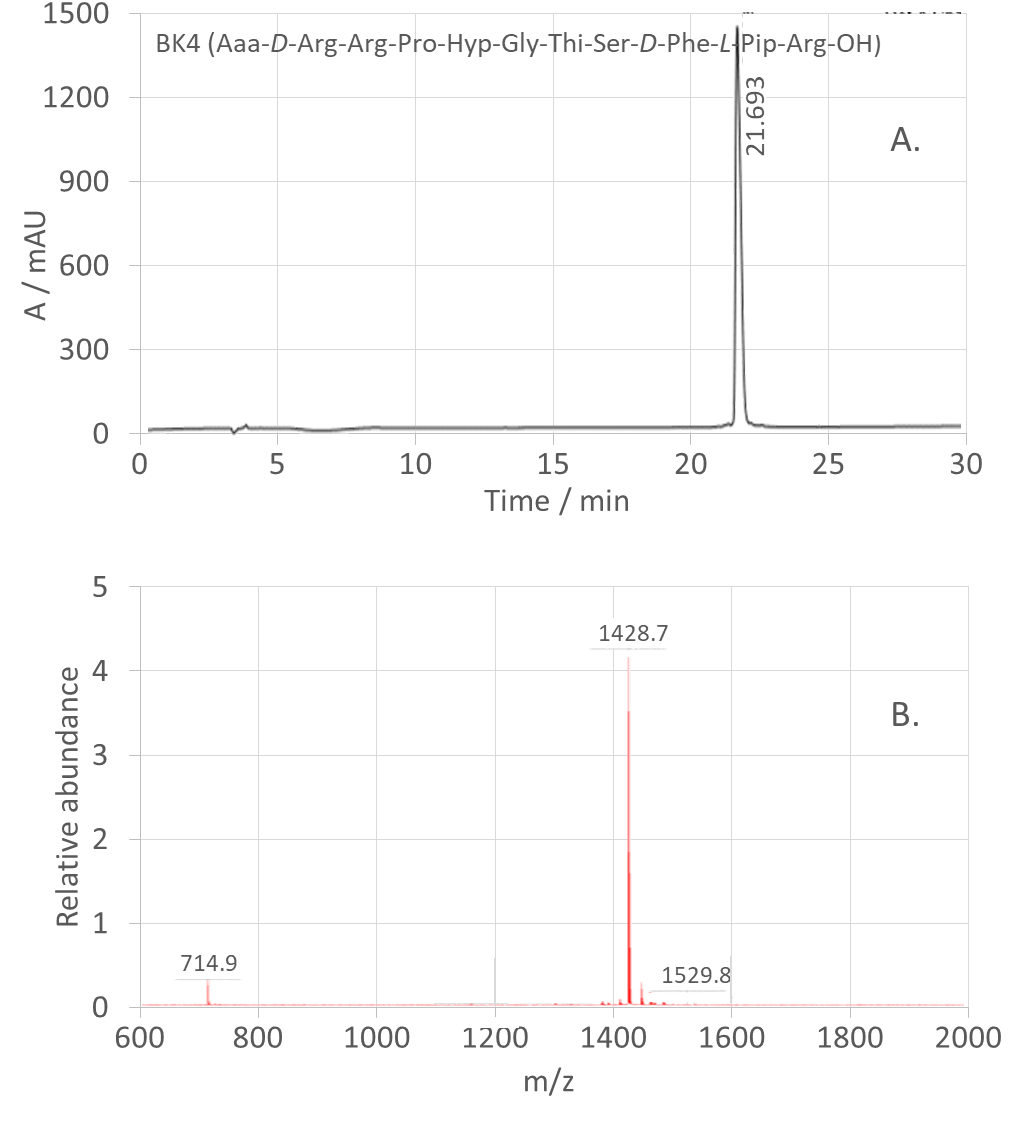
***

**Figure 5S.** The RP-HPLC analysis (A.) and mass spectrum ([M+H]^+^ calculated 1428.1, found 1428.7) (B.) of BK4 peptide.

**
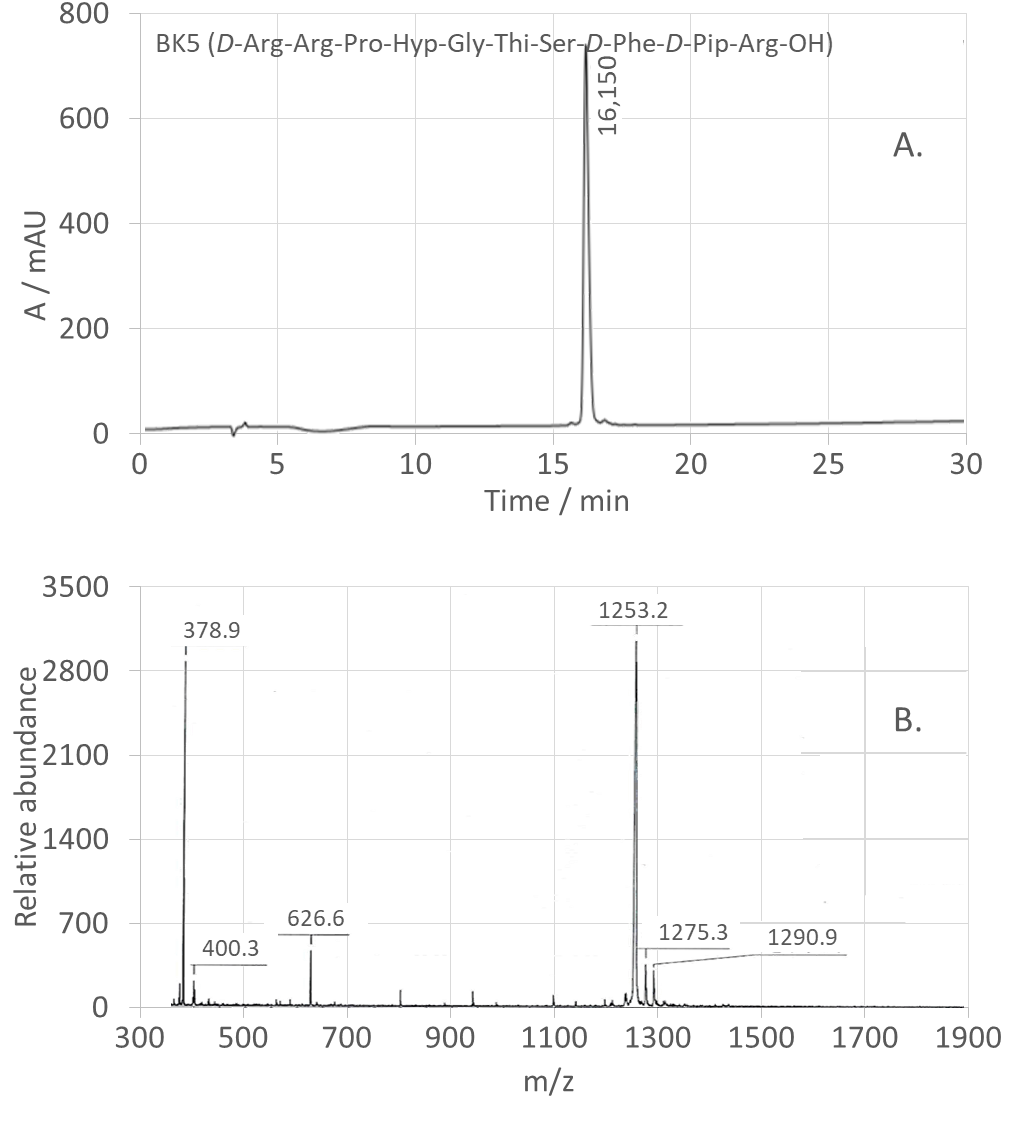
**

**Figure 6S.** The RP-HPLC analysis (A.) and mass spectrum ([M+H]^+^ calculated 1253.1, found 1253.0) (B.) of BK5 peptide.

**
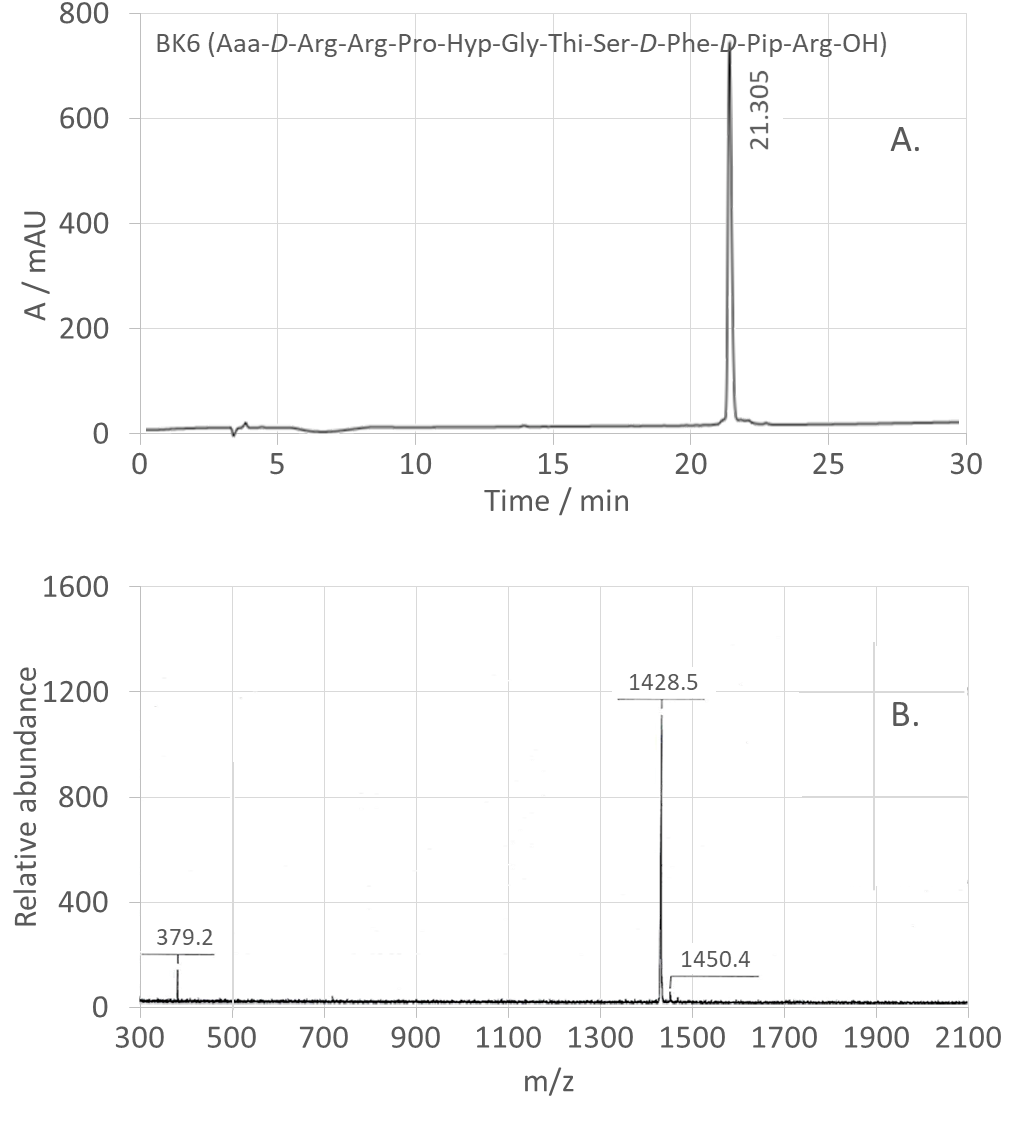

Figure 7S.** The RP-HPLC analysis (A.) and mass spectrum ([M+H]^+^ calculated 1428.1, found 1428.5) (B.) of BK6 peptide.


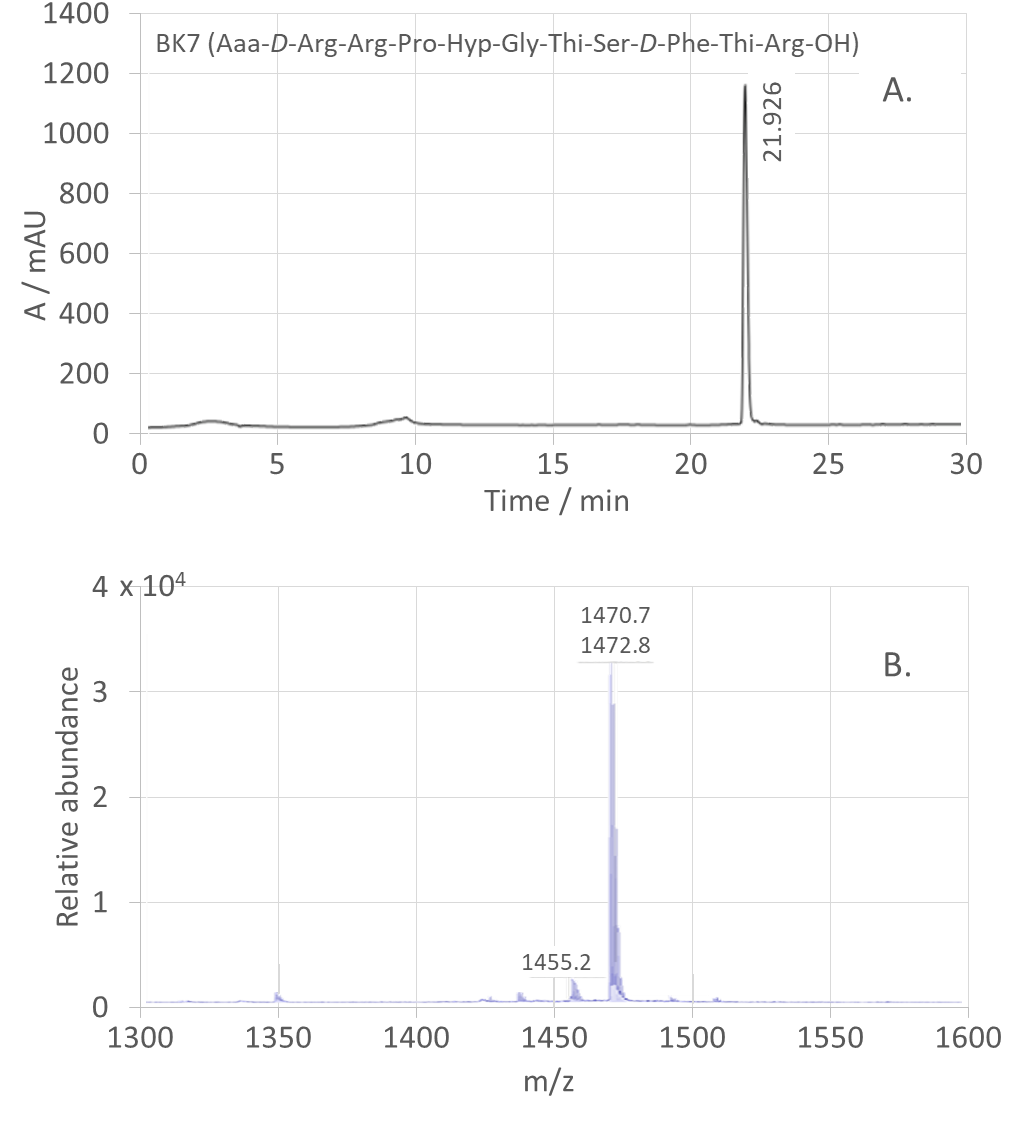


**Figure 8S.** The RP-HPLC analysis (A.) and mass spectrum ([M+H]^+^ calculated 1470.3, found 1470.1) (B.) of BK7 peptide.


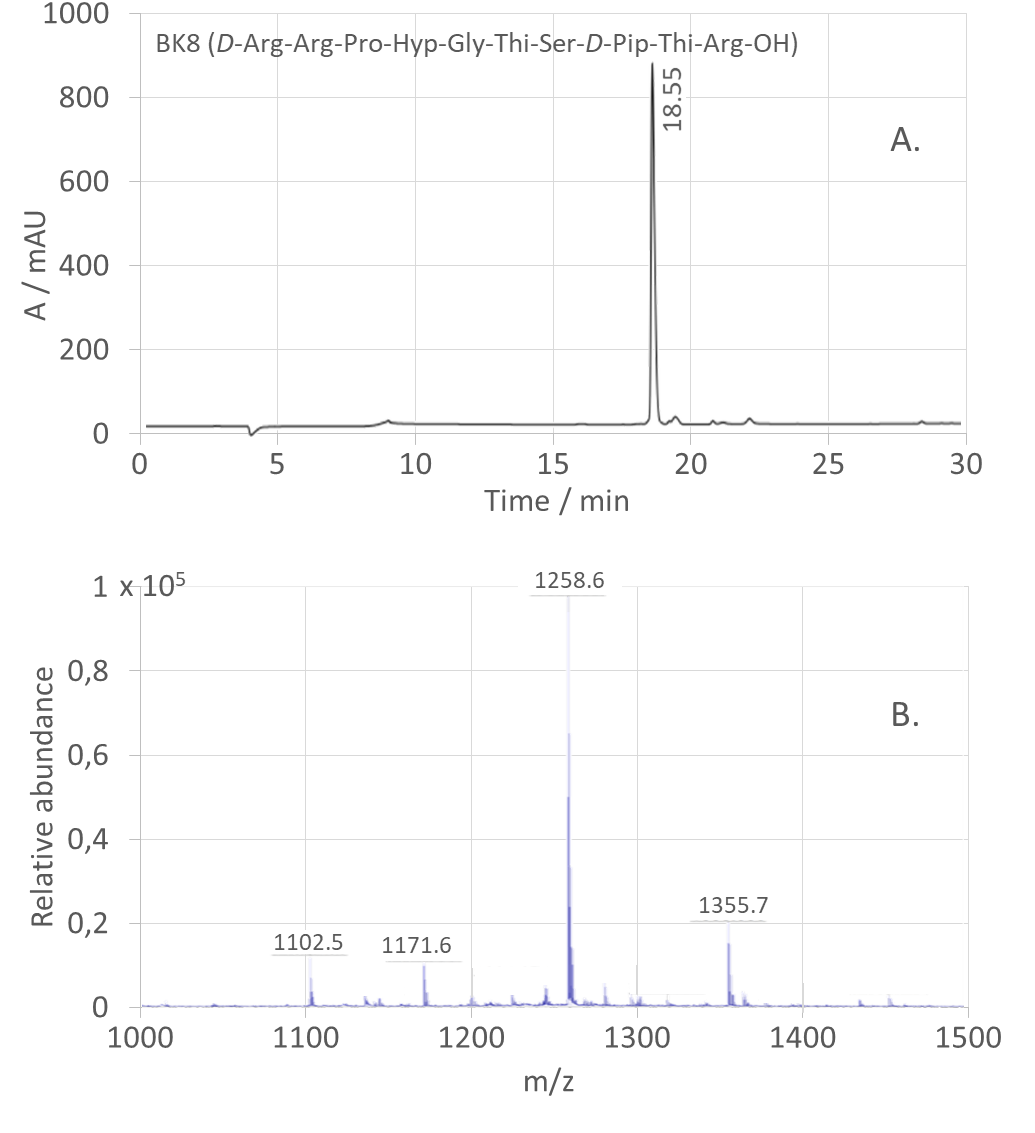


**Figure 9S.** The RP-HPLC analysis (A.) and mass spectrum ([M+H]^+^ calculated 1258.9, found 1258.6) (B.) of BK8 peptide.


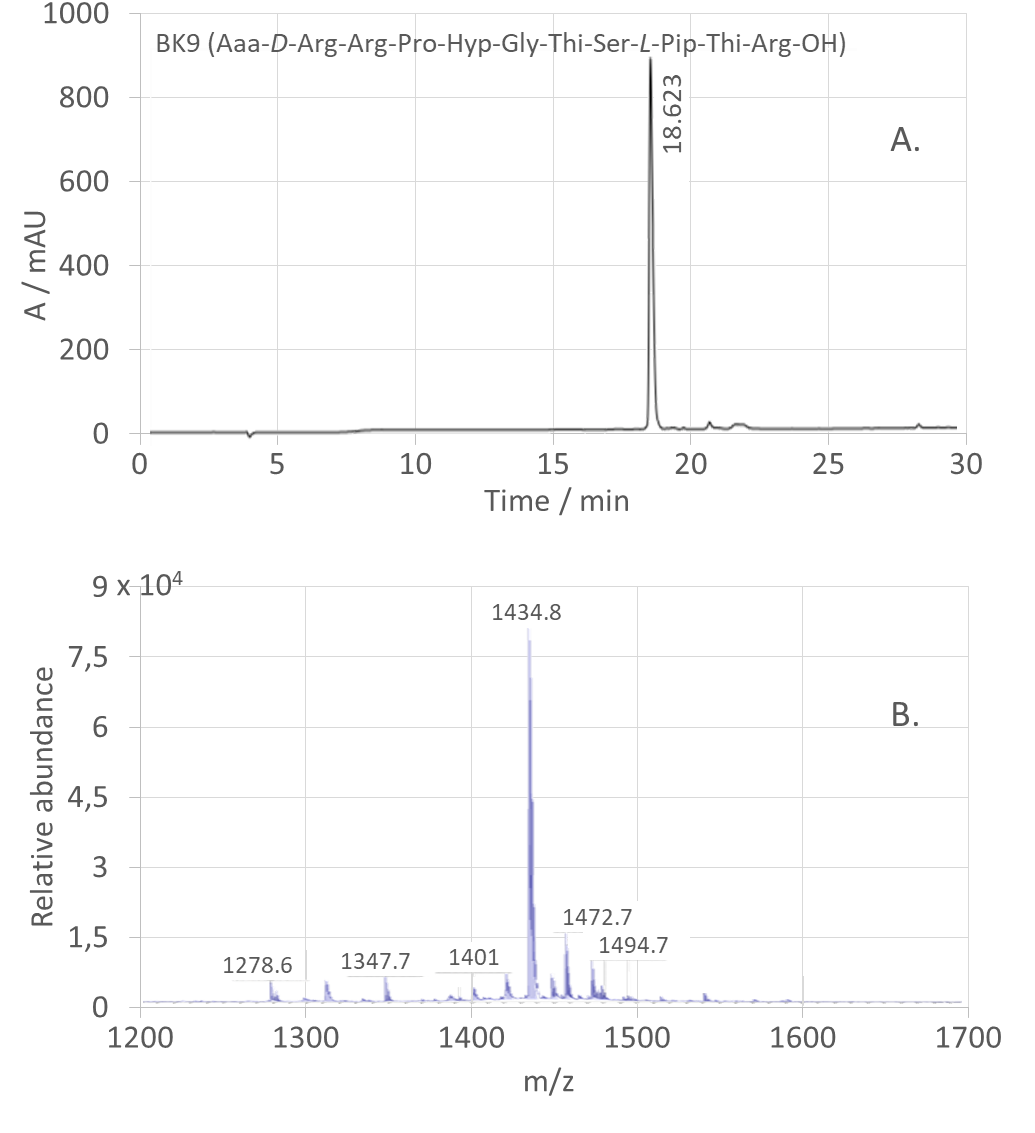


**Figure 10S.** The RP-HPLC analysis (A.) and mass spectrum ([M+H]^+^ calculated 1433.9, found 1434.8) (B.) of BK9 peptide.

***
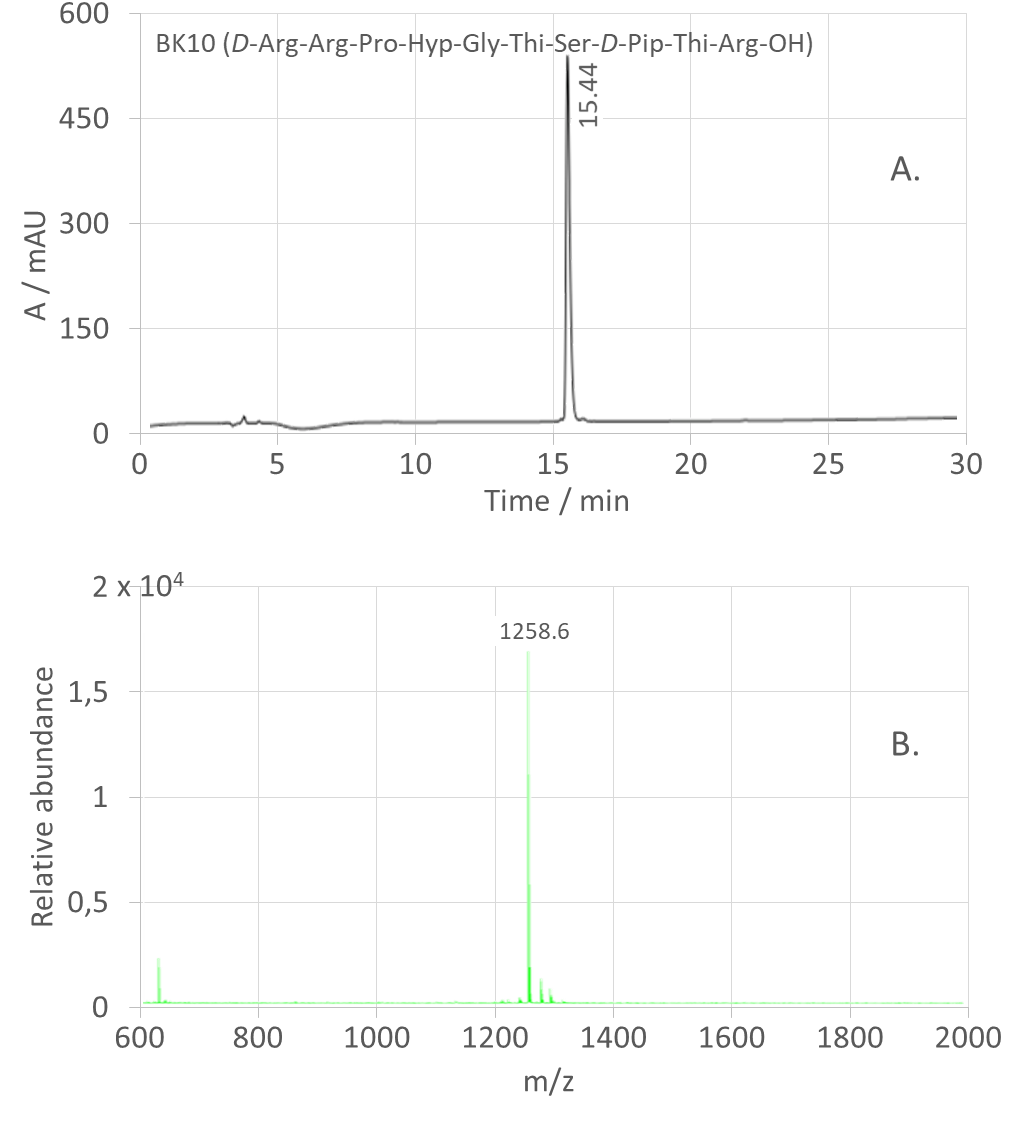
***

**Figure 11S.** The RP-HPLC analysis (A.) and mass spectrum ([M+H]^+^ calculated 1258.9, found 1258.6) (B.) of BK10 peptide.


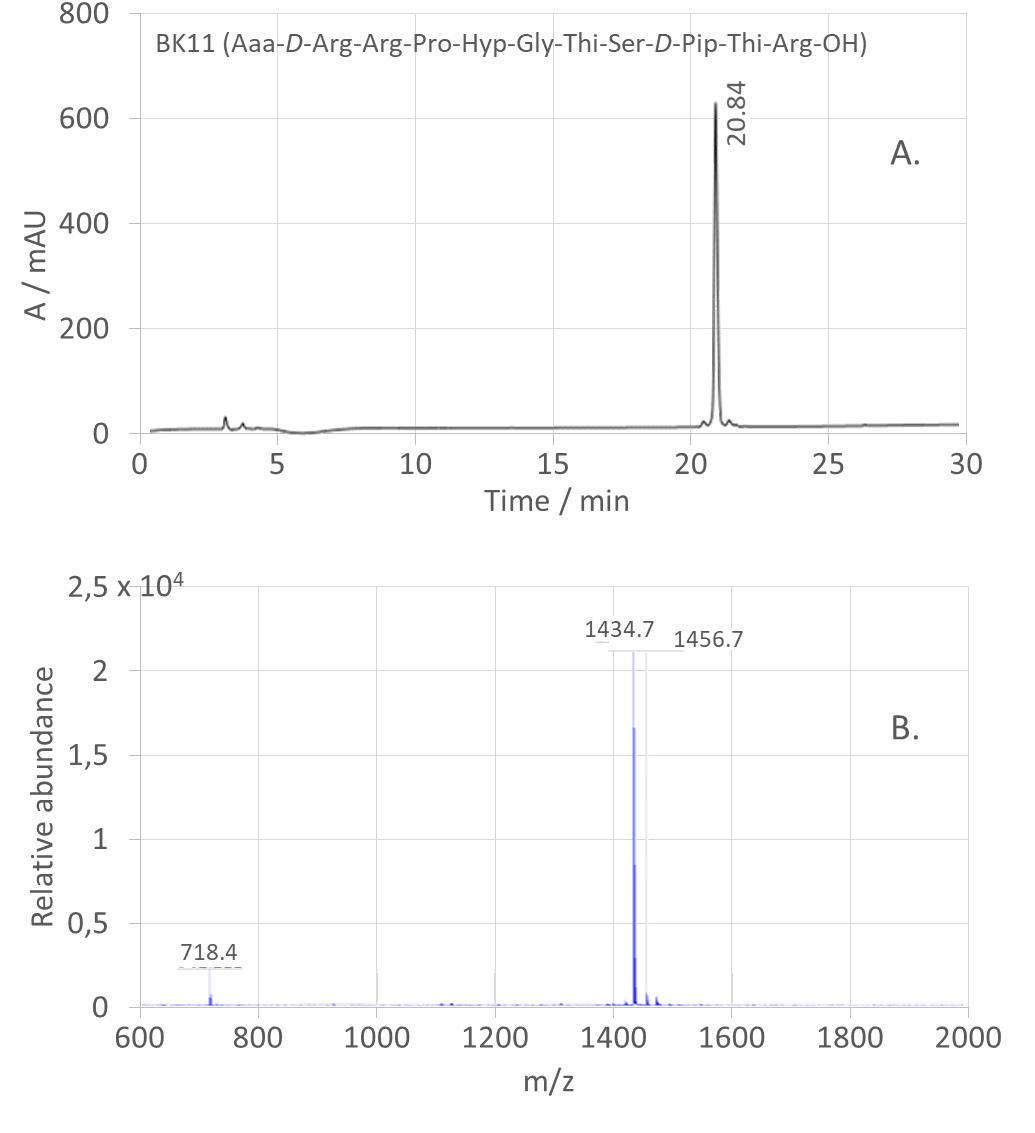


**Figure 12S.** The RP-HPLC analysis (A.) and mass spectrum ([M+H]^+^ calculated 1433.9, found 1434.7) (B.) of BK11 peptide.

**
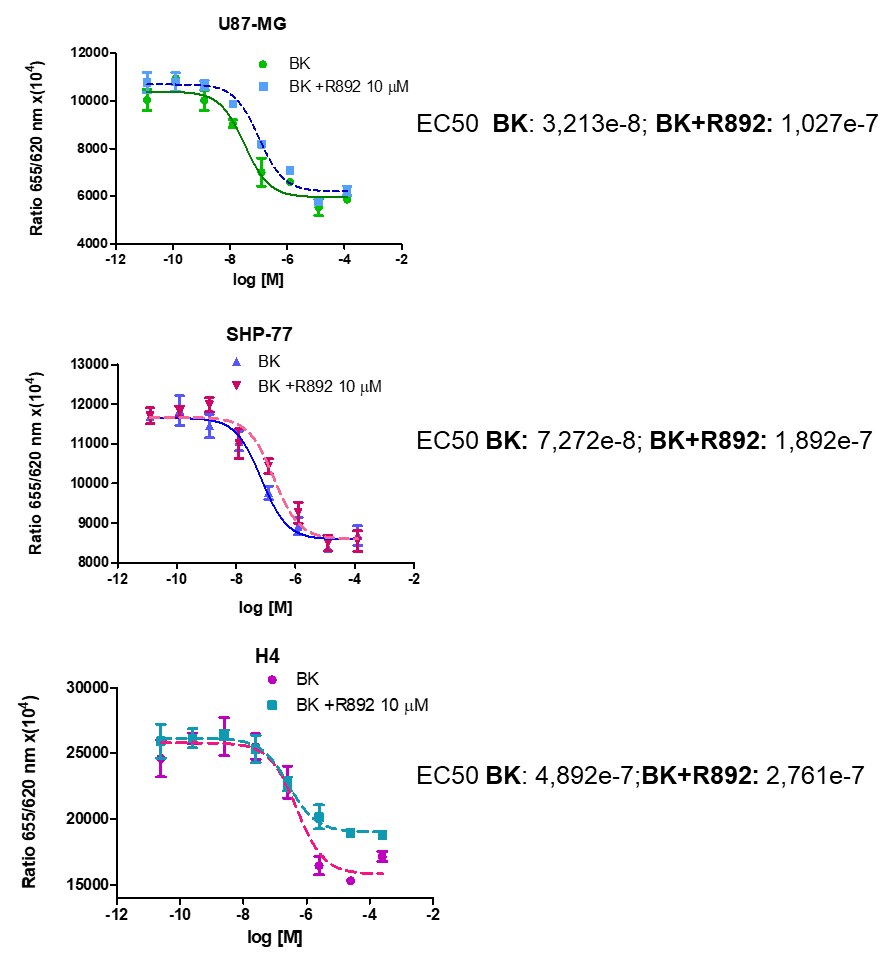
**

**Figure 13S.** The results present a shift of the bradykinin (BK) dose-response curve after treatment with the specific B1R antagonist R892 at a concentration of 10 µM in the cancer cell lines: U87-MG, SHP-77, and H4. The highest effect was observed in the U87-MG cell line where EC50 was shifted over 3 times, for SHP-77 shift was 2,5x and for H4 cells was the lowest least than 2x. All three cell lines expressed the functional protein B1R.

**
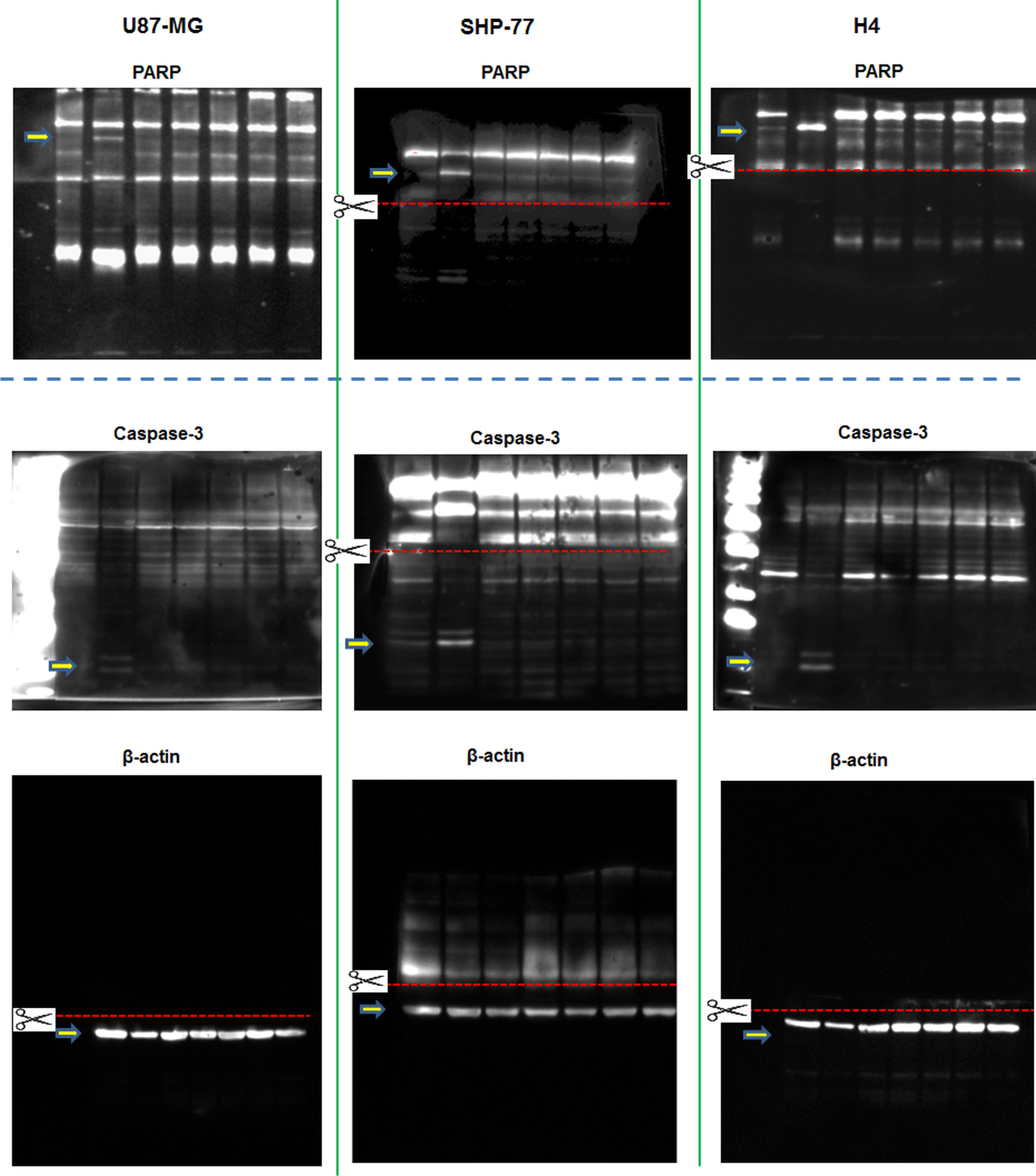
Figure 14S.** The effects of B1R antagonists and cisplatin on the expression levels of caspase-3 and PARP cleavage in cell lines U87-MG, SHP-77, and H4 were determined by Western blotting. **1.** control cells; **2.** cisplatin (25 μM); **3.** BK7 (50 μM); **4.** BK3 (50 μM); **5.** BK4 (50 μM); **6.** BK10 (50 µM); **7.** BK11 (50 μM). Representative Western blot images showing original, unprocessed versions.
